# Identification, characterization, and rescue of CRISPR/Cas9 generated wheat *SPO11‐1* mutants

**DOI:** 10.1111/pbi.13961

**Published:** 2022-12-10

**Authors:** Lucy Hyde, Kim Osman, Mark Winfield, Eugenio Sanchez‐Moran, James D. Higgins, Ian R. Henderson, Caroline Sparks, F. Chris H. Franklin, Keith J. Edwards

**Affiliations:** ^1^ School of Biological Sciences, Life Sciences University of Bristol Bristol UK; ^2^ School of Biosciences University of Birmingham Birmingham UK; ^3^ Department of Genetics and Genome Biology University of Leicester Leicester UK; ^4^ Department of Plant Sciences University of Cambridge Cambridge UK; ^5^ Rothamsted Research Hertfordshire UK

**Keywords:** wheat CRISPR/Cas9, genome editing, meiosis, *SPO11‐1*, wheat transformation

## Abstract

Increasing crop yields through plant breeding is time consuming and laborious, with the generation of novel combinations of alleles being limited by chromosomal linkage blocks and linkage‐drag. Meiotic recombination is essential to create novel genetic variation via the reshuffling of parental alleles. The exchange of genetic information between homologous chromosomes occurs at crossover (CO) sites but CO frequency is often low and unevenly distributed. This bias creates the problem of linkage‐drag in recombination ‘cold’ regions, where undesirable variation remains linked to useful traits. In plants, programmed meiosis‐specific DNA double‐strand breaks, catalysed by the *SPO11* complex, initiate the recombination pathway, although only ~5% result in the formation of COs. To study the role of *SPO11‐1* in wheat meiosis, and as a prelude to manipulation, we used CRISPR/Cas9 to generate edits in all three *SPO11‐1* homoeologues of hexaploid wheat. Characterization of progeny lines shows plants deficient in all six *SPO11‐1* copies fail to undergo chromosome synapsis, lack COs and are sterile. In contrast, lines carrying a single copy of any one of the three wild‐type homoeologues are phenotypically indistinguishable from unedited plants both in terms of vegetative growth and fertility. However, cytogenetic analysis of the edited plants suggests that homoeologues differ in their ability to generate COs and in the dynamics of synapsis. In addition, we show that the transformation of wheat mutants carrying six edited copies of *SPO11‐1* with the *TaSPO11‐1B* gene, restores synapsis, CO formation, and fertility and hence opens a route to modifying recombination in this agronomically important crop.

## Introduction

Increasing crop yields through plant breeding is a time consuming and laborious process; it typically takes up to 10 years to develop a new crop variety and bring it to market. Current breeding methods involve targeting novel, advantageous allelic combinations, whilst segregating away alleles for undesirable traits through meiotic recombination. However, breeders are currently hampered in their ability to develop novel combinations of alleles as the available breeding germplasm is limited by the presence of chromosomal linkage blocks and the effects of linkage‐drag (Wijnker and de Jong, 2008).

Meiotic recombination is essential both to create novel genetic variation in progeny via the shuffling of parental alleles, and to generate viable gametes via the alignment, synapsis, and subsequent segregation of homologous chromosomes (Mercier *et al*., 2015). During meiosis, homologous chromosomes closely align and eventually become tightly linked (synapsed) along their length by the proteinaceous synaptonemal complex (SC) (Page and Hawley, 2004). In plants, chromosome axis morphogenesis and synapsis can be monitored cytologically by immunolocalization of ASY1, which marks the unsynapsed chromosome axes (Armstrong *et al*., 2002), and ZYP1, the transverse filament protein of the SC (Higgins *et al*., 2005). In hexaploid wheat, synapsis begins near the chromosome ends (telomeres) which cluster into a restricted region of the nucleus during early prophase I (Osman *et al*., 2021; Sepsi *et al*., 2017). As synapsis progresses, the ASY1 signal becomes gradually replaced by a linear ZYP1 signal until synapsis is complete in late prophase I (Osman *et al*., 2021). This chromosome remodelling is coordinated with the exchange of genetic information between homologous chromosomes via homologous recombination at crossover (CO) recombination sites. This is determined by the frequency and physical position of COs which are cytologically detectable as chiasmata at meiotic metaphase I (Lambing and Heckmann, 2018).

In most eukaryotes, CO frequency is low with one to three COs per homologue pair (Mercier *et al*., 2015). In addition, the distribution of COs along chromosomes is non‐random and occurs at favoured chromosome sites, often referred to as ‘hotspots’, frequently located in sub‐telomeric regions in plant genomes (Higgins *et al*., 2014; Lambing *et al*., 2017; Osman *et al*., 2021). For example, Saintenac *et al*. (2009) report that on wheat chromosome 3B, 90% of COs occur in distal regions representing only about 40% of the physical length of the chromosome. Choulet *et al*. (2014), in a study of 305 individual plants, report an even greater bias suggesting that all COs occurred in only 13% of the chromosome arm. Bias in CO distribution creates the problem of linkage‐drag in the recombination‐cold regions proximal to the centromere where undesirable variation cannot be readily separated from useful traits. It would be desirable to be able to manipulate the frequency and distribution of COs to generate novel allelic combinations (Wijnker and de Jong, 2008).

Crossovers arise through the repair of programmed DNA‐double strand breaks (DSBs) via homologous recombination (HR) during the first meiotic division (Mercier *et al*., 2015). In land plants, meiotic DSBs are formed via a heterotetrameric catalytic complex comprising a *SPO11‐1*/*SPO11‐2* heterodimer and an MTOPVIB homodimer (Roberts *et al*., 2016; Vrielynck *et al*., 2016). *SPO11* is an evolutionarily conserved type II topoisomerase‐like enzyme, which is homologous to the A subunit of topoisomerase VI in the archaeal species *Sulfolobus shibatae* (Bergerat *et al*., 1997). Most eukaryotes possess a single copy of *SPO11*, although multiple *SPO11* orthologues have been identified in some plant species. For instance, *Arabidopsis* possesses three orthologues (Hartung and Puchta, 2001) whilst rice has five (An *et al*., 2011). In both cases, only *SPO11‐1* and *SPO11‐2* are essential for meiosis (Fayos *et al*., 2020; Hartung *et al*., 2007). *SPO11‐1* and *SPO11‐2* have also been identified in hexaploid bread wheat (*Triticum aestivum*) and *SPO11‐2* has been found to be essential for DSB formation (Benyahya *et al*., 2020).

*SPO11* activity generates numerous DSBs of which a small proportion are repaired as COs. For example, in *Arabidopsis SPO11* catalyses the formation of around 200 DSBs of which typically only 10 become COs (Chelysheva *et al*., 2007; Sanchez‐Moran *et al*., 2007). In most animal and plant species, high levels of DSBs are essential for the alignment and synapsis of homologous chromosomes during meiotic prophase I, which are in turn a prerequisite for CO formation (Zickler and Kleckner, 2015). In some organisms, stable CO numbers are robustly maintained even when DSB numbers change considerably, a phenomenon known as CO homeostasis (Martini *et al*., 2006). In plants, the effects of CO homeostasis appear to be more limited. Analysis of maize inbred lines indicated that the number of COs is correlated with DSB frequency (Sidhu *et al*., 2015). In *Arabidopsis*, transgenic lines with a 30%–40% reduction in meiotic DSB levels formed proportionally fewer COs (Xue *et al*., 2018), and notably, COs in pericentromeric regions were substantially reduced. Given its key role in DSB formation, the *SPO11* complex, or one of its components, would appear as a logical target for the manipulation of COs (Yelina *et al*., 2022). The potential of this approach has been previously demonstrated in budding yeast using *Spo11* fused to a range of DNA recognition modules (Sarno *et al*., 2017).

Although hexaploid wheat possesses three homoeologous genomes, at meiosis it behaves as a diploid due to the presence of the *Ph1* and *Ph2* loci on chromosomes 5B and 3D, respectively (Griffiths *et al*., 2006; Serra *et al*., 2021). There are three homoeologous copies of *SPO11‐1* in wheat, one on chromosome 5 of each of the three subgenomes (*TaSPO11‐1‐5A*, *TaSPO11‐1‐5B*, and *TaSPO11‐1‐5D*). The homoeologues are highly similar and, respectively, encode proteins of 387, 386 and 387 amino acids, all of which are reported to be functional (Da Ines *et al*., 2020). Consequently, our experimental approach was based on the assumption that it would be necessary to modify all three homoeologues (i.e., all 6 copies of the gene) to obtain a biological effect. Thus, here we report the generation of CRISPR‐Cas9 loss‐of‐function edits in all three *TaSPO11‐1* homoeologues. This has enabled us to analyse the meiotic role of *SPO11‐1* and create the possibility of manipulating CO number and position in future wheat breeding programmes. We report a detailed characterization of these gene‐edited lines and the effect of the edited alleles on DSB formation, synapsis, and CO formation during meiosis. Further, using *TaSPO11‐1B* as a transgene, we have rescued the sterile phenotype of plants triple‐edited for all 3 *SPO11‐1* homoeologues. We demonstrate that this construct complements the edits and restores chromosome synapsis, CO formation, and fertility, albeit with varying levels of efficiency. These results, and the resources developed during this study, should provide the basis to test the potential of modified *SPO11‐1* constructs to influence the number and distribution of COs along chromosome arms of bread wheat.

## Results

### Generation of hexaploid wheat *SPO11‐1* edits

**Figure 1:**
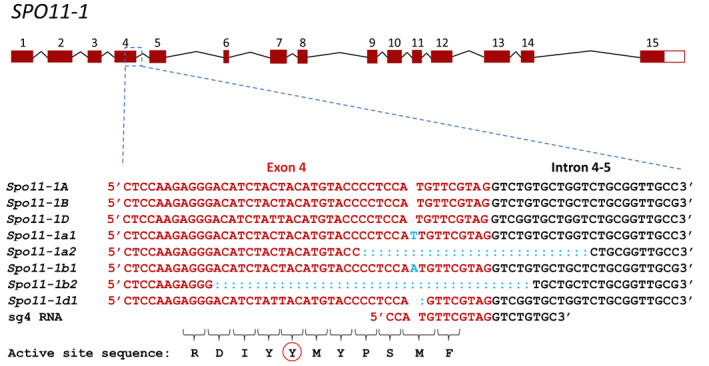
Gene structure of *SPO11‐1* showing exons (numbered) as red boxes and introns as black lines. Represented below the diagram are the sequences across the boundary between exon 4 (red type face) and intron 4‐5 (black type face) of wild type alleles and the edited alleles from Plant 22; the 1 bp inserts are shown in sky blue. The amino acid sequence of the active site is shown at the bottom of the diagram; the tyrosine of the active site is circled in red.

Sprink and Hartung (2014) have previously shown that *SPO11* genes generate a complex pattern of alternatively spliced transcripts. We were therefore concerned that editing events that did not target the active site, that is, the site responsible for generating DSBs, might fail to generate null alleles. Therefore, we specifically designed guide RNAs (sg4 5′GCACAGACCTACGAACATGG3′ and sg6 5′GGAGAGGACGTCCGTGCCGA3 ′) to flank the sequences coding for the catalytic tyrosine (Y129 in *SPO11‐1A* and *SPO11‐1D*, and Y128 in *SPO11‐1B*) of the protein's active site (Figure 1; File S1; Figure S1). The activity of the two guides was confirmed by co‐transformation (as a single plasmid), with wheat optimized Cas9 (Zhang *et al*., 2019), into wheat protoplasts (File S1; Figure S2). Co‐transformation of wheat variety Cadenza with the same Cas9 and guides generated 46 independent T_0_ transgenic lines. Examination of these plants, via amplification across the region covered by the two guide RNAs, revealed that only plants 22 and 26 had edits within the *SPO11‐1* target region; interestingly, edits were found only around the sg4 guide RNA and not the sg6 guide. Plant 26 had a single edit in the B genome copy of *SPO11‐1*, and plant 22 had edits in five of its six *SPO11‐1* genes: two different edits of both the A and B genome copies and a single edit of the D genome copy (Table 1; Figure 1). Consequently, plant 22 was used in all subsequent analyses. Characterization of subsequent generations of plant 22 failed to find evidence for any further editing events in either selfed or crossed lines; however, we did specifically select for lines lacking the Cas9/sgRNA construct.

**Table 1:** Summary of the five *spo11‐1* edits found in plant 22

Homoeologue	Edit	Allele name	Predicted result
*SPO11‐1A*	1 bp insertion	*spo11‐1a1*	Truncated protein
	27 bp deletion	*spo11‐1a2*	Truncated protein
*SPO11‐1B*	1 bp insertion	*spo11‐1b1*	Truncated protein
	38 bp deletion	*spo11‐1b2*	Complete protein minus active site
*SPO11‐1D*	1 bp deletion	*spo11‐1d1*	Truncated protein

### Characterization of edited alleles

**Figure 2:**
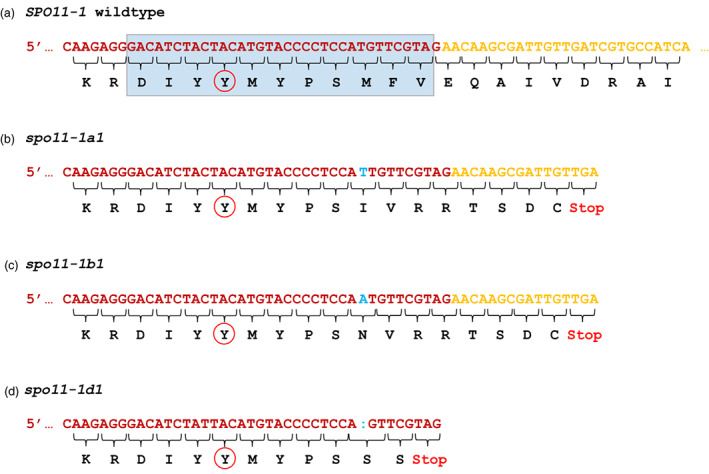
Wild type and edited nucleotide and amino acid sequences spanning the junction between exon 4 (red font) and exon 5 (yellow font). a) Wild type sequence; the light blue box defines the active site of the DNA binding domain, the active site tyrosine is circled in red. b) and c) The 1 bp insertions (blue font) in *spo11-1a1* and *spo11-1b1* result in a protein truncated in exon 5. d) The 1 bp deletion (blue colon) in *spo11-1d1* results in a protein truncated in exon 4.

*TaSPO11‐1* is a complex gene with 15 exons (Figure 1), and gene edits might be spliced out of the mature mRNA. To investigate this, we extracted total RNA from immature ears of edited plants and used RT‐PCR and Sanger sequencing to examine the resultant transcripts. Using the appropriate primers (File S2), we amplified *SPO11‐1* transcripts from RNA obtained from immature ears of various plants carrying different combinations of wild‐type and edited alleles (Table 1). Sanger sequencing of the amplified transcripts indicated that each of the edited alleles produces a distinct transcript: alleles *spo11‐1a1* and *spo11‐1b1*, both 1 bp insertions, result in a full‐length transcript containing a frameshift leading to a predicted truncated protein terminating 12 amino acids downstream of the catalytic tyrosine (Figure 2b,c); allele *spo11‐1d1*, a 1 bp deletion, produces a transcript resulting in a predicted protein that is truncated six amino acids downstream of the tyrosine active site (Figure 2d).

**Figure 3:**
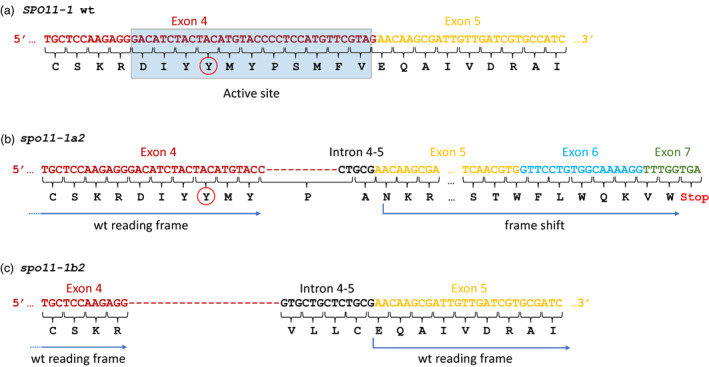
Nucleotide and amino acid sequences spanning the exon 4 to exon 5 junction of wild type and edited alleles of *SPO11‐1*. a) Wild type sequence showing the active site (blue box). b) Sequence of edited allele *spo11‐1a2* which carries a 27 bp deletion with respect to wild type: 15 bp have been removed from the 3’ end of exon 4 and replaced with 5 bp from intron 4‐5 resulting in a frameshift that creates a stop codon at the beginning of exon 7. c) Sequences of edited allele *spo11‐1b2* which carries a 38 bp deletion with respect to wild type: 34 bp, spanning the whole of the active site, have been removed from the 3’ end of exon 4 as have the first 4 bp from the 5’ end of intron 4‐5); 13 bp from intron 4‐5, immediately after the edit, have become incorporated into the new coding sequence which maintains the wild type reading frame through exon 5; as a consequence, eleven amino acids spanning the active site are deleted from *spo11‐1b2* and replaced with four novel amino acids. The exon sequences are colour coded and labelled; the sequence from intron 4‐5 is in black type face.

Allele *spo11‐1a2* has a 27 bp deletion spanning the splice junction between exon 4 and intron 4–5 that results in the loss of 15 bp from the 3′ end of exon 4 but the gain of 5 bp (CTGCG) from intron 4–5 immediately after the deletion; this restores the GT splice site but results in a frameshift in reading across exons 5 and 6 and, ultimately, a stop codon in exon 7 (Figure 3b).

Allele *spo11‐1b2* carries a 38 bp deletion spanning the active site and the 5′ splice junction of intron 4–5 (Figure 3c). The original reading frame is maintained and a predicted full‐length protein, missing the active site but maintaining all the other *SPO11‐1* functional domains, is produced.

### Triple‐edited *SPO11‐1* plants are sterile

Although plant 22 carried only a single *SPO11‐1* wild‐type allele (*SPO11‐1D*), it was phenotypically normal at all stages of vegetative growth, including ear development, and generated a similar number of tillers to wild‐type Cadenza (c. eight tillers per plant). On selfing, the plant generated a similar number of viable seeds (350) to wild‐type Cadenza (average for 10 plants = 340). Genotyping of T_1_ seedlings (*n* = 96) showed them to carry all possible combinations of *SPO11‐1* alleles, including 20 lines with edits in all six copies of the gene. All 96 plants, irrespective of their genotype, showed normal vegetative growth. Interestingly, all plants with at least one wild‐type allele were fertile. By contrast, plants carrying only edited alleles, regardless of the combination in which these were found, were always sterile and frequently exhibited a distorted ear morphology. Overall, the segregation pattern of the various wild‐type and edited alleles showed no significant variation from the expected pattern (File S1; Table S1).

**Figure 4:**
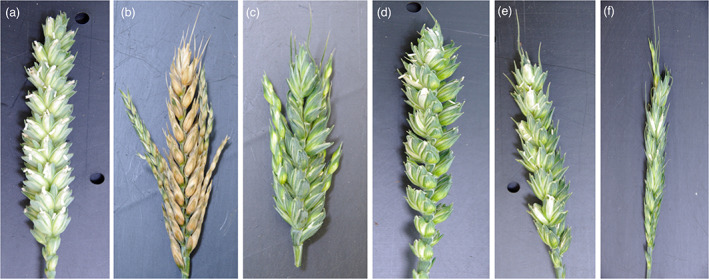
Spikes from wild‐type Cadenza and triple‐edited plants (aabbdd). (a) Cadenza wt. (b and c) Triple For Review Only edited plants (aabbdd) showing distorted ears with no seed set. (d, e, and f) Triple edited plants carrying the *SPO11‐1B* transgene; these lines had seed set 10%–50% that of wt. All images are of ears 21 days post‐anthesis.

Plant 22 was also crossed with wild‐type Cadenza, and the F1s selfed to generate several lines carrying diverse combinations of wild‐type and edited alleles: that is, plants carrying from 0 to 6 edited alleles, along with plants carrying a single wild‐type allele, *SPO11‐1A*, *SPO11‐1B*, or *SPO11‐1D* (hereafter referred to as plants *Aabbdd*, *aaBbdd*, and *aabbDd*, respectively). Again, in all cases (*n* = 300), plants carrying six edited alleles (regardless of allele combination) showed normal vegetative growth but produced no seed; as for the selfed lines, frequently, but not always, such plants had distorted ears (Figure 4b,c). In contrast, plants carrying a single wild‐type allele, regardless of which, were fully fertile and showed no ear distortion.

### Cytological analysis of plants

We studied meiosis in a plant homozygous for edits in all three *SPO11‐1* homoeologues, hereafter referred to as *aabbdd*, which is homozygous for the 27 bp deletion in the A homoeologue, the 38 bp deletion in the B homoeologue, and the 1 bp deletion in the D homoeologue. We also studied plants containing a single copy of a wild‐type allele (plants *Aabbdd*, *aaBbdd*, and *aabbDd*). Meiotic progression in these plants was compared to that in wild‐type Cadenza.

#### Chromosome synapsis does not occur in triple‐edited plants (aabbdd)

**Figure 5:**
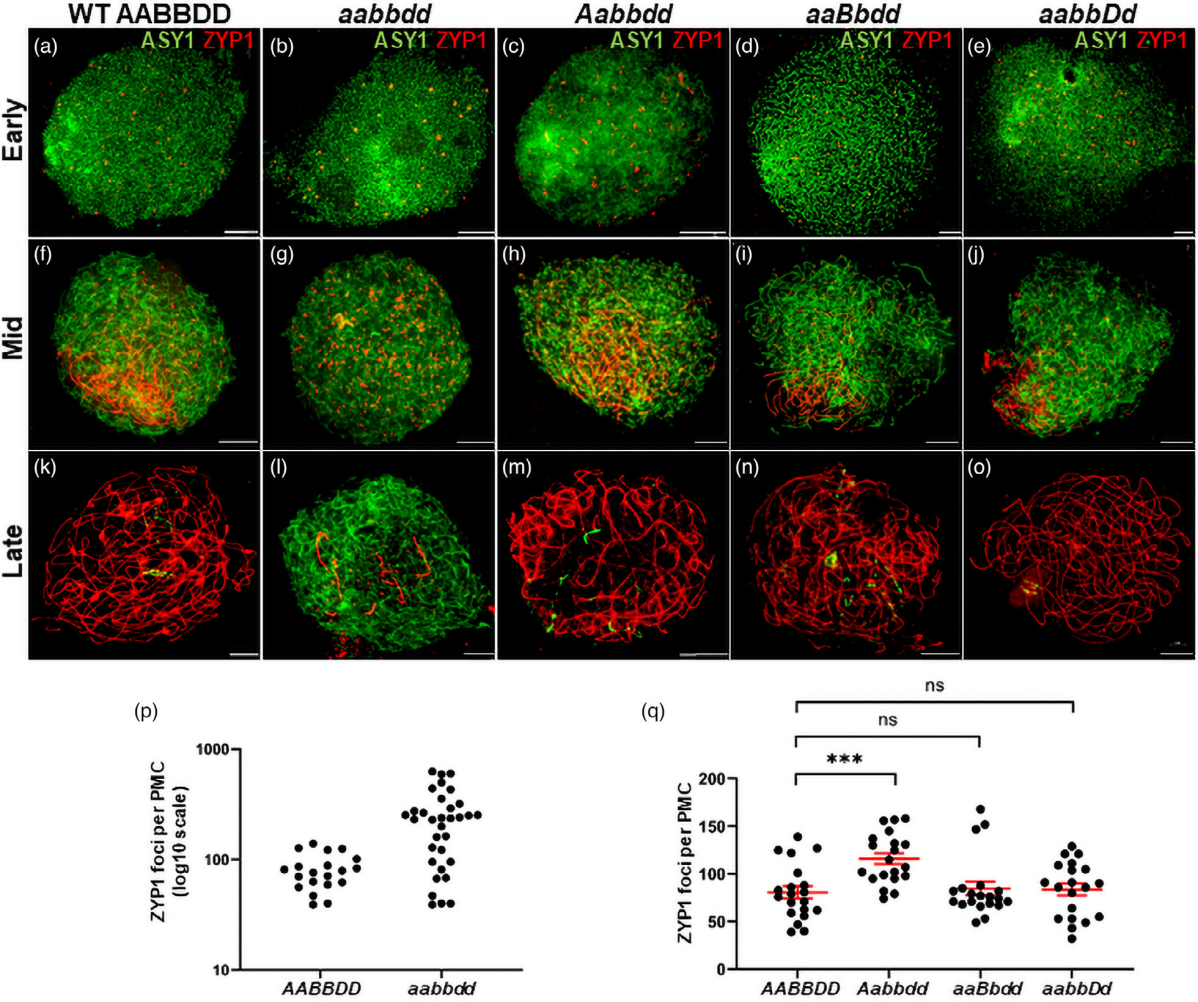
Immunolocalisation of ASY1 and ZYP1 in pollen mother cells (PMCs) from wild‐type Cadenza, a *spo11‐1* triple‐edited plant (aabbdd), and plants carrying a single copy of a wild*type allele. (a–e) Early prophase I: all edited lines appear similar to the wild type with characteristic regions of intense ASY1 signal which mark the sub‐telomeres where axis development is most advanced (Osman *et al*., 2021), and presynaptic ZYP1 foci. (f–j) Mid prophase I: wild type and lines with a single wild type allele begin to synapse and form polarized linear SC (f, h–j); aabbdd accumulates numerous ZYP1 foci but does not form linear SC (g). (k–o) Late prophase I: wild type and lines with a single wild‐type allele are fully synapsed with just a slight trace of ASY1 remaining (k, m–o); aabbdd forms a few short linear stretches of ZYP1 but remains largely unsynapsed (l). (p) Counts of presynaptic ZYP1 foci in wild type and ZYP1 foci in aabbdd in early‐mid prophase I. (q) Counts of presynaptic ZYP1 foci in wild type and single copy wild type lines. In all images, the scale bar represents 10 μm. In q, bars indicate the mean and SE; *** denotes statistical significance at the 0.1% level and ns denotes no significant difference.

During axis linearisation in early prophase I, *aabbdd* plants appeared similar to wild type, exhibiting a distinctive region of intense ASY1 signal, corresponding to clustered telomeres, and characteristic presynaptic ZYP1 foci (Figure 5a,b). However, they did not enter a normal zygotene or begin to form linear SC (Figure 5f,g). Instead, they continued to accumulate axis‐associated ZYP1 foci, forming up to fourfold the number observed in wild type prior to the onset of SC extension (maximum 626, *n* = 32 v 139, *n* = 20) (Figure 5g,p). In late prophase I, when chromosomes in the wild type fully synapsed, *aabbdd* remained unsynapsed forming only a few short stretches of linear ZYP1 (Figure 5k,l). In contrast, lines *Aabbdd*, *aaBbdd*, and *aabbDd* all behaved similarly to the wild type (Figure 5k,m–o).

Interestingly, a more detailed examination indicated that the dynamics of synapsis in *Aabbdd* might subtly differ from that seen in wild type and both *aaBbdd* and *aabbDd*. On average, *Aabbdd* accumulated significantly more presynaptic ZYP1 foci than the wild type before any linear SC extension was observed (mean = 116.0 ± 5.93 v 80.7 ± 6.53, *n* = 20, *P* = 2.85 × 10^−4^) (Figure 5q). On the other hand, the number of presynaptic ZYP1 foci in *aaBbdd* and *aabbDd* were not significantly different from the wild type (mean = 84.8 ± 7.20, *P* = 0.68 and 83.7 ± 6.47, *P* = 0.75 respectively, *n* = 20) (Figure 5q).

#### Triple‐edited plants (aabbdd) fail to form chiasmata

**Figure 6:**
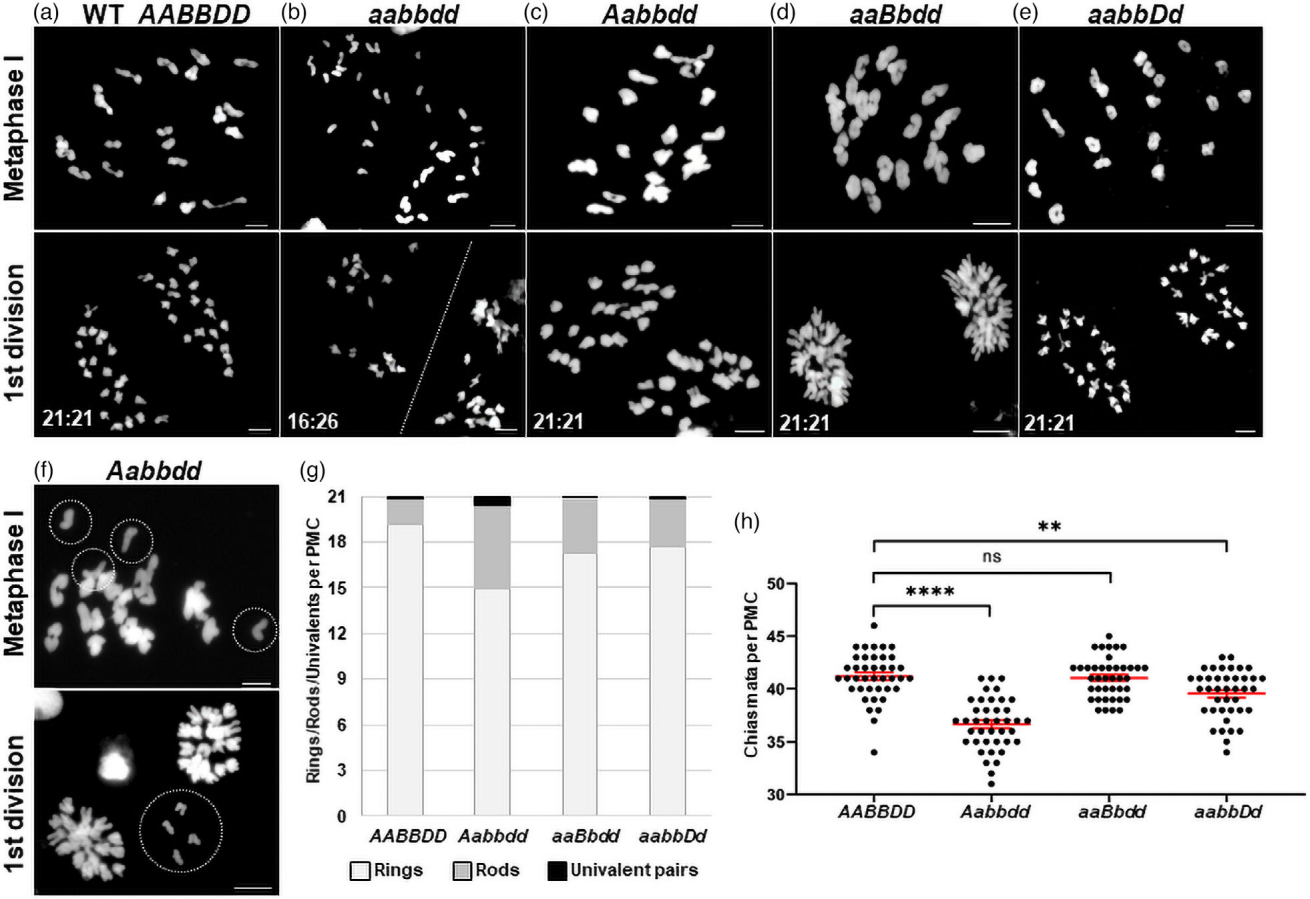
4′,6‐diamidino‐2‐phenylindole (DAPI) stained meiotic chromosomes from PMCs from wild‐type Cadenza, *spo11‐1* triple‐edited plants (aabbdd), and plants carrying a single copy of the wild‐type gene. (a–e top panel): Chromosome spreads at metaphase I. Cadenza wild type (a) and plants with a single wild‐type allele (c–e) formed 21 bivalents; aabbdd plants (b) formed 42 univalents. (a–e bottom panel): at first division, wild‐type Cadenza (a) and lines with a single wild‐type allele (c–e) showed normal segregation; segregation in the aabbdd plant (b) was unbalanced—the plane of separation is shown as a dotted line. (f) Aabbdd PMC with achiasmate chromosomes; (f top panel) two achiasmate chromosomes present as two pairs of univalents (circled) at metaphase I; (f bottom panel) lagging chromosomes at first division (circled). One univalent pair presents as four sister chromatids due to premature separation. (g) Mean proportion of univalent pairs and ring and rod bivalents in a single PMC (*n* = 40). (h) Chiasma frequency per PMC with mean and SE bars (*n* = 40). **** and ** denote statistical significance at the 0.01% and 1% levels respectively and ns denotes no significant difference. In all images, the scale bar = 10 μm.

As expected, most pollen mother cells (PMCs) in wild‐type Cadenza (87.5%, *n* = 40) formed 21 bivalents at metaphase I and, consequently, went on to segregate two cohorts of 21 chromosomes at the first meiotic division (Figure 6a, upper and lower panels, respectively). The remaining PMCs contained 20 bivalents and two univalent chromosomes due to the lack of a chiasma in one homologous pair (not shown). In contrast to the wild type, PMCs from *aabbdd* plants presented 42 univalents at metaphase I (*n* = 50), indicating a complete absence of chiasmata, resulting in unbalanced segregation at the first division (Figure 6b, upper and lower panels, respectively). As in wild‐type plants, most PMCs from lines containing a single wild‐type allele (*Aabbdd*, *aaBbdd*, and *aabbDd*), presented 21 bivalents at metaphase I and balanced segregation at the first meiotic division (Figure 6c–e). However, a more detailed analysis revealed some interesting differences between the *SPO11‐1* homoeologues regarding their ability to maintain chiasmata. In wild‐type Cadenza, the number of achiasmate chromosomes during meiosis was relatively low: 12.5% of nuclei (*n* = 40) contained just one achiasmate chromosome which presented as univalents at metaphase I. Achiasmate chromosomes were also observed in *Aabbdd*, *aaBbdd*, and *aabbDd* plants, but in *aaBbdd* and *aabbDd* these were no more frequent than in wild type (7.6% and 12.5% of PMCs, respectively; *n* = 40). However, in *Aabbdd*, 30% of PMCs had one achiasmate chromosome (*n* = 40) with a further 15% containing two achiasmate chromosomes (Figure 6f, upper panel), a situation not observed in wild type, *aaBbdd* or *aabbDd*. Nevertheless, although we observed examples of unattached, ‘lagging’ chromosomes in *Aabbdd* (Figure 6f, lower panel), most nuclei segregated with the normal 21:21 ratio (85%, *n* = 20), consistent with the high level of fertility displayed by this line.

There were also differences between lines with regard to the number of chiasmata formed during meiosis. Line *Aabbdd* formed significantly fewer chiasmata than wild type (mean per PMC = 36.7 ± 2.5 v 41.2 ± 2.2, *n* = 40, *P* = 6.25 × 10^−13^) (Figure 6h). Mean chiasma frequency was also reduced in *aaBbdd* and *aabbDd* but less markedly than in *Aabbdd* and, in the case of *aaBbdd*, the reduction was not significantly different to wild‐type (*aaBbdd*, 41.1 ± 2.19, *n* = 40, *P* = 0.75; *aabbDd*, 39.6 ± 2.3, *n* = 40, *P* = 1.67 × 10^−3^). These differences were reflected in the number of rod bivalents (a consequence of chiasma(ta) occurring in only one chromosome arm) and ring bivalents (chiasmata in both chromosome arms) observed in the different lines. *Aabbdd* had threefold more rod bivalents than wild type (5.4 v 1.8 per PMC, *n* = 40), whilst lines *aaBbdd* and *aabbDd* had more than wild type, but fewer than *Aabbdd* (3.7 and 3.2 rods per PMC, respectively; *n* = 40) (Figure 6).

### *SPO11‐1* and DMC1 localization in triple‐edited plants (aabbdd)

**Figure 7:**
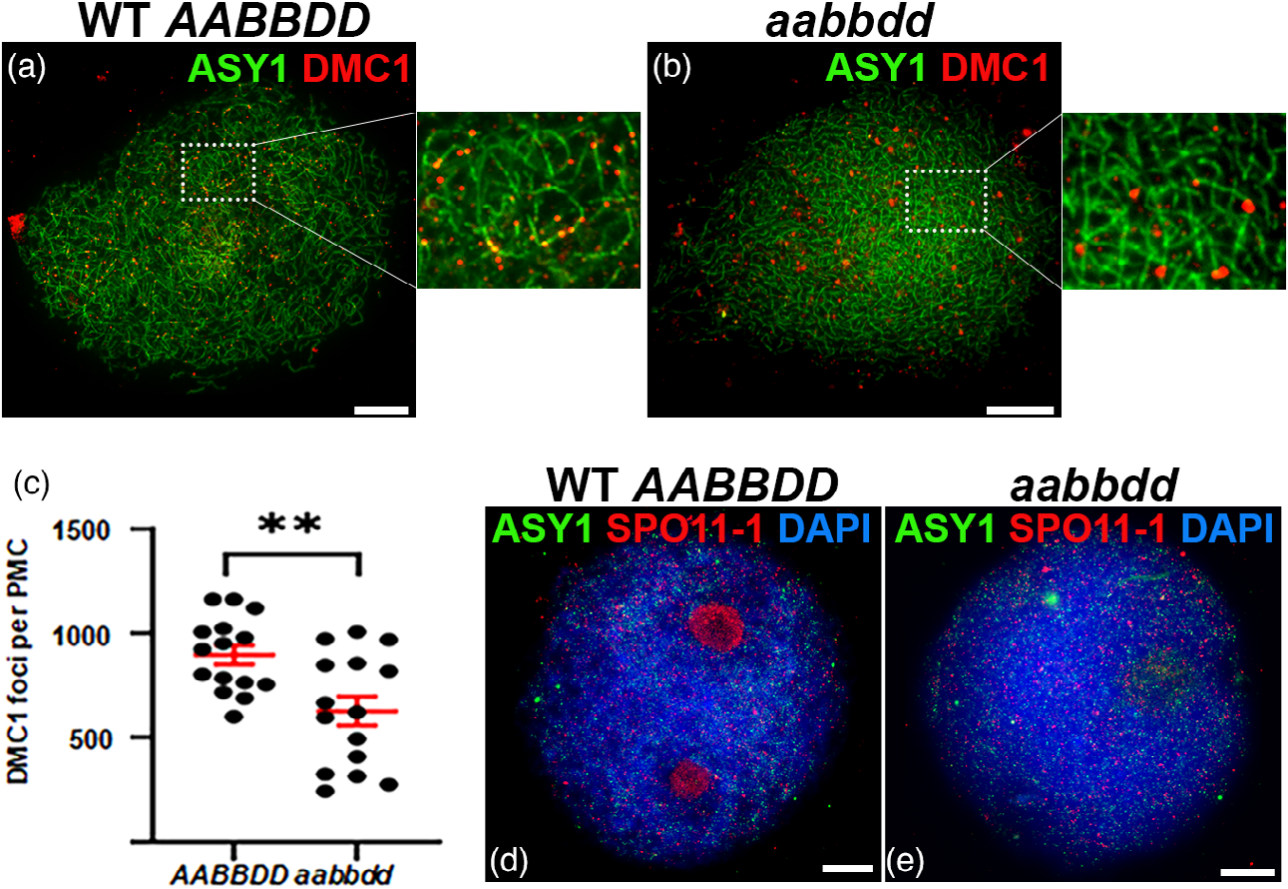
Immunolocalisation of *SPO11‐1* and DMC1 in PMCs from wild type and a *spo11‐1* triple mutant plant (aabbdd). Images show a single z‐frame from the middle of a nucleus. (a and b) Dual localization of DMC1 and ASY1 in wild type (a) and aabbdd (b) in early prophase I when the ASY1 axis signal is linear. (c) Counts of DMC1 foci in early prophase I. Bars indicate mean and SE, ** denotes statistical significance at the 1% level. (d and e) Dual localization of *SPO11‐1* and ASY1 at the G2/prophase I transition when ASY1 foci first appear. DNA is stained with 4′,6‐diamidino‐2‐phenylindole (DAPI). Scale bars represent 10 μM.

In previous plant studies, the formation of *SPO11*‐dependent meiotic DSBs has routinely been assessed indirectly using immunocytochemistry (Chelysheva *et al*., 2007; de Muyt *et al*., 2007; Ku *et al*., 2020; Sanchez‐Moran *et al*., 2007). For example, foci of DMC1, a recombinase involved in the early stages of recombination‐mediated DSB repair, were used to estimate the number of meiotic DSBs in studies of rice *spo11‐1* and *spo11‐2* mutants and a wheat *spo11‐2* mutant (Benyahya *et al*., 2020; Fayos *et al*., 2020). We therefore examined DMC1 localization in the *spo11‐1* triple mutant in early prophase I when the ASY1 axis signal appeared linear. At this stage, wild‐type plants formed numerous DMC1 foci along the chromosome axis (Figure 7a,c) consistent with previous analysis in Cadenza (Osman *et al*., 2021). Surprisingly, axis‐associated DMC1 foci were also observed in *aabbdd* plants, indicating only a modest yet significant decrease to ~70% compared to wild type (mean = 627 ± 71 v 896 ± 46, *n* = 15, *P* = 3.6 × 10^−3^) (Figure 7b,c). Notably, foci in *aabbdd* appeared very heterogeneous in size and shape with many appearing much bigger than in wild type (Figure 7a,b) but we did not discriminate between morphological types during foci counts.

In view of the presence of DMC1 foci, we wondered whether *aabbdd* plants might still be capable of forming a *SPO11*‐like complex. RT‐PCR analysis and Sanger sequencing had demonstrated that the *spo11‐1b2* allele of *aabbdd* plants produces a full‐length transcript, simply lacking 38 bp of sequence spanning the active site and the 5′ splice junction of intron 4–5 and maintaining the open reading frame of the wild‐type allele (see Figure 3c). This transcript is predicted to encode a full‐length protein, lacking the active site but maintaining all other SPO11‐1 functional domains and thus potentially capable of interacting with other components of the DSB machinery and being recruited to chromatin. To investigate this, we carried out *SPO11‐1* immunolocalization in *aabbdd* using an antibody directed against a short peptide sequence spanning the junction of exons 9 and 10 which was expected to detect the predicted protein product of the *spo11‐1b2* allele but not the predicted truncated protein products encoded by the edited A and D alleles. In the wild type, *SPO11‐1* forms numerous foci throughout the chromatin early in meiosis at the transition of G2 into prophase I (Figure 7d). Similar *SPO11‐1* foci were observed throughout the chromatin in *aabbdd* plants (Figure 7e) suggesting that the *spo11‐1b2* transcript is translated into a protein product, presumably inactive, which can be recruited to chromatin. At this stage ASY1 also formed numerous foci throughout the chromatin but there appeared no particular colocalization between the two proteins in either wild type or *aabbdd* plants. In the wild type, the *SPO11‐1* antibody also prominently stained the nucleoli, a phenomenon that has been noted for other antibodies and other plant species and it has been suggested that the nucleolus may act as a reservoir for sequestering meiotic proteins, as it does for cell cycle proteins (Visintin and Amon, 2000). Nucleolar staining was not observed in *aabbdd* but may simply reflect reduced protein abundance relative to the wild type.

In summary, the evidence suggests that an inactive form of *SPO11‐1* and the downstream recombinase, DMC1, can be recruited to the chromosomes in *aabbdd* plants but DMC1 foci are reduced in number, many of them appear morphologically abnormal and chromosome synapsis cannot occur. This is consistent with a defect in normal DSB formation in this line.

### Complementation of *aabbdd* plants

We transformed *aabbdd* embryos with an 8.55 kb genomic fragment containing the entire *SPO11‐1‐5B* gene (3.17 kb promoter, 3.92 kb structural gene, and 1.46 kb 3′ untranslated region) and in total, eight transgene‐positive *aabbdd* plants were generated. However, plants B133wa2 and B133wa3 were derived from a single embryo, as were B12p2se1 and B12p2se2, and, hence, considered as only two events. On growing the six independent lines to anthesis, five generated fertile ears, although they showed varying degrees of seed set, and one, B12p2se1/se2, was sterile showing no seed set (Table 2).

**Table 2:** Lines positive for the transgene (*SPO11‐1B*)

Transformant	Genotype	Fertility	Notes
B12p2sb2	*aaBbdd*	Fertile	Heterozygote for endogenous B *Spo11‐1*
B12p2sd1	*aabbdd*	Fertile	Fertility/seed set similar to wild‐type Cadenza
B133wa2/wa3	*aabbdd*	Fertile	Two lines derived from the same callus; they had similar levels of fertility/seed set (c. 50% of wt Cadenza)
B12p2sd2	*aabbdd*	?	One ear in T0 set seed, rest sterile, all progenies were sterile
B12p3sa1	*aabbdd*	Fertile	Low fertility/seed set (c.10% of wild‐type Cadenza)
B135Ba4	*aabbdd*	Fertile	Fertility/seed set (c. 30% of wild‐type Cadenza)
B12p2se1/se2	*aabbdd*	Sterile	No seed set

#### Cytological analysis of transformed plants

Four *aabbdd* transgene‐positive plants with varying degrees of restored fertility were analysed cytologically. In addition, as controls, two tissue culture‐derived lines that lacked the transgene were included in the analysis: one control carried six wild‐type alleles (designated con‐*AABBDD*) and the other carried only edited alleles (designated con‐*aabbdd*).

**Figure 8:**
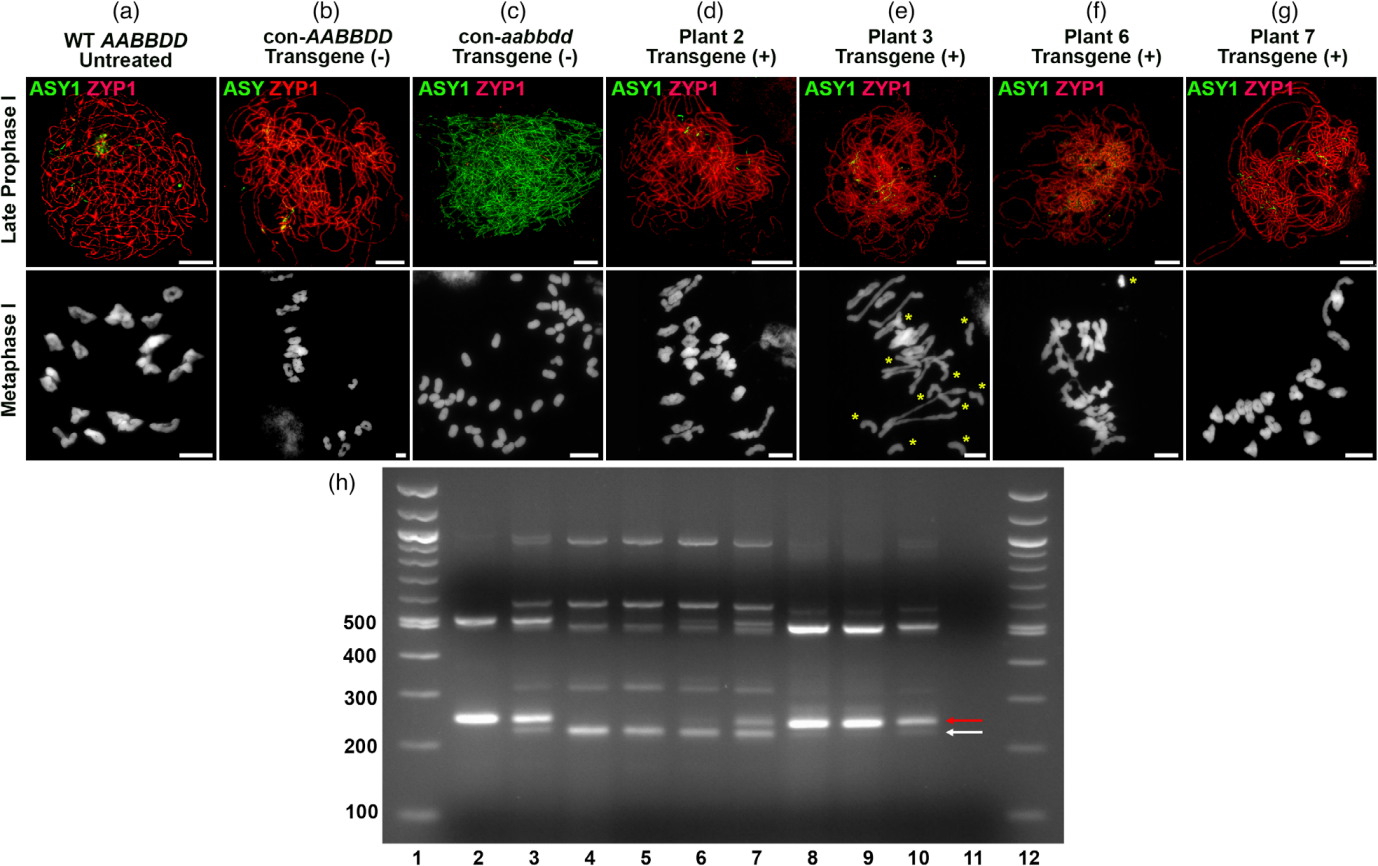
The presence of the *SPO11‐1B* transgene complements the *spo11‐1* triple mutant (aabbdd), restoring chromosome synapsis, chiasma formation, and fertility. (a–g top panel): Immunolocalisation of ASY1 and ZYP1 in PMCs at late prophase I. Cadenza wild type (a), tissue culture derived con‐AABBDD (b) and four transgene‐positive *spo11‐1* triple‐edited plants (d–g) showed linear ZYP1 signal throughout the nucleus indicating synapsis; tissue culture derived con‐aabbdd plants (c) failed to synapse and ASY1 remained marking the chromosome axes. (a–g bottom panel) DAPI‐stained chromosome spreads at metaphase I. Most Cadenza wild type (a) and con‐AABBDD (b) PMCs formed 21 bivalents; all con‐aabbdd PMCs (c) contained 42 univalents due to a failure to form chiasmata; all four transgene‐positive *spo11‐1* triple‐edited plants (d–g) contained numerous bivalents indicating a restoration of chiasma formation, albeit with varying levels of efficiency. Yellow asterisks indicate univalent chromosomes in (e) and (f). Scale bars represent 10 μM. (h) Semi‐quantitative RT‐PCR of RNA derived from immature ears of various nontransgenic and transgenic wheat lines; amplification performed using B genome‐specific primers flanking the edited region of *SPO11‐1*. Lanes 1 and 12 = 100 bp ladder; lane 2 = wild type Cadenza; lane 3 = Cadenza heterozygous for the 38 bp edit; lane 4 non‐transgenic aabbdd; lane 5 aabbdd transformed with Bar; lanes 6 and 7 = aabbdd transgene +ve (plant 6); lane 8 = aabbdd transgene +ve (plant 2); lane 9 = aabbdd transgene +ve (plant 3); lane 10 = aabbdd transgene +ve (plant 7); and lane 11 = water control. The lower, white arrow indicates the edited B genome‐specific *spo11‐1* transcript; the upper, red arrow indicates the wild type B genome‐specific *SPO11‐1* transcript encoded by either the endogenous allele (lanes 2 and 3) or the transgene (lanes 3, 6–9). Higher MW bands represent alternatively spliced transcripts generated from either the endogenous gene/transgene or the edited B allele.

Immunolocalisation of ASY1 and ZYP1 was carried out to examine the ability of the *aabbdd* transgene‐positive lines to undergo chromosome synapsis. Both tissue culture‐derived controls behaved as expected: *con‐AABBDD* fully synapsed and, by late prophase I, linear ZYP1 signal was observed along the entire length of the chromosomes (Figure 8b). In contrast, *con‐aabbdd* did not synapse and ASY1 remained marking the axes of the unsynapsed chromosomes until late prophase I (Figure 8c). In the four transgene‐positive *aabbdd* lines, ZYP1 formed along the length of all chromosomes indicating that all were capable of full synapsis (Figure 8d–g).

Metaphase I spreads from con‐*AABBDD* were similar to wild type having 21 bivalents in most PMCs (Figure 8a,b; Table 3); only 3.8% of nuclei contained a univalent pair (*n* = 26). In contrast, con‐*aabbdd* failed to form chiasmata and all PMCs contained 42 univalents at metaphase I (*n* = 40) (Figure 8c).

**Table 3:** Number of univalent pairs and seed set in transformed plants

Plant	Freq. nuclei with one or more univalent pairs (%)	Comments	Fertility (seeds per ear)
Cadenza WT	12.5 (*n* = 40)	Max no. univalent pairs = 1	42.2 (*n* = 24)
*con‐AABBDD*	3.8 (*n* = 26)	Max no. univalent pairs = 1	40.1 (*n* = 11)
*con‐aabbdd*	100 (*n* = 40)	All chromosomes present as univalents	0 (*n* = 10)
2	13.9 (*n* = 144)	Max no. univalent pairs = 1	40.6 (*n* = 14)
3	41.0 (*n* = 39)	Max no. univalent pairs = 5	19.1 (*n* = 27)
6	Not assessed	Very few metaphase I nuclei	8.9 (*n* = 11)
7	19.7 (*n* = 71)	Max no. univalent pairs = 1	18 (*n* = 11)

All four transgene‐positive *aabbdd* lines were able to form chiasmata, confirming successful complementation by the *SPO11‐1B* transgene (Figure 8d–g). However, the efficiency of chiasma restoration varied between the lines. Line 2 showed the highest efficiency with only 13.9% of nuclei exhibiting a univalent pair (*n* = 144) compared to 12.5% (*n* = 40) in the wild type (Figure 8d and Table 3). In lines 3 and 7 (Figure 8e,g and Table 3), the percentage of nuclei with at least one univalent pair was 41.0% (*n* = 39) and 19.7% (*n* = 71), respectively, with up to five univalent pairs per nucleus observed in line 3. In the case of line 6, we were only able to identify four metaphase I‐like nuclei despite repeated attempts using a range of anther sizes. Therefore, although we could confirm the presence of chiasmata (Figure 8f), we were unable to assess univalent frequency. In this line, most of the PMCs observed were at prophase I suggesting that there might be a delay in meiotic progression, but this was not investigated further. The variation in cytological data between the four transgene‐positive lines was broadly consistent with their fertility (Table 3). That is, the mean number of seeds set per spike in wild‐type Cadenza and *con‐AABBDD* was 40.1 (*n* = 11) and 42.2 (*n* = 24), respectively. Line 2, which exhibited very few univalent pairs during meiosis, had a good seed set; 40.6 (*n* = 14). For lines 3 and 7, this figure was 19.1 (*n* = 27) and 18.0 (*n* = 11), respectively, whilst in line 6 it was only 8.9 (*n* = 11). As expected, *con‐aabbdd*, which lacked chiasmata, failed to produce seed.

To investigate if the cytological and phenotypic data from the various transgenes was related to the level of transgene expression, we carried out semi‐quantitative PCR on RNA from immature ear tissues using primers specific for the B genome transcript of *SPO11‐1* (Figure 8h). In all transgenic lines examined the level of B‐genome transcript correlated with the cytological and phenotypic data, such that none or low levels of transgene transcript correlated with sterility whereas higher levels of transcript correlated with higher levels of fertility.

## Discussion

Meiotic recombination in plants is initiated by the formation of DSBs catalysed by a heterotetrameric protein complex composed of a heterodimer of *SPO11‐1* and *SPO11‐2* and a homodimer of MTOPVIB (Vrielynck *et al*., 2016). Failure of DSB formation is known to lead to defects in meiotic progression (Grelon *et al*., 2001). That is, alignment and synapsis of homologous chromosomes are defective and Cos fail to form leading to unbalanced segregation into nascent gametes and, ultimately, to sterility (Stacey *et al*., 2006). As a first step in the manipulation of recombination, we investigated the role of *SPO11‐1* in meiosis in hexaploid bread wheat. To do this, we used Cas9 to disrupt the active site of all six copies of the gene. Although we were successful in generating a single plant carrying edits in all three *SPO11‐1* homoeologues, overall the frequency of editing observed was low (2/46 or 4.3%). Surprisingly, editing events occurred solely around the sg4 guide RNA; in no case did we observe editing around the sg6 guide RNA. This is in contrast with what we observed in protoplasts where both guide RNAs produced edits (File S2), suggesting that the performance of guide RNAs in protoplasts is not necessarily an accurate indicator of their efficacy in transgenic plants. A comparison of our editing frequency with those of Zhang *et al*. (2019), who used the same Cas9 construct but for different targets, suggests that the choice of target, wheat variety, and transformation procedure may all play a part in determining the overall efficiency of editing events recovered. Mutants of *spo11‐1* and *spo11‐1* silenced lines have been characterized in *Arabidopsis* (Grelon *et al*., 2001; Hartung *et al*., 2007; Puizina *et al*., 2004; Stacey *et al*., 2006), rice (Fayos *et al*., 2020; Yu *et al*., 2010) and maize (Ku *et al*., 2020), and *spo11* mutants are well‐characterized in animals and fungi (Baudat *et al*., 2000; Celerin *et al*., 2000; Keeney *et al*., 1997). However, to the best of our knowledge, this is the first report of *spo11‐1* mutants in wheat. In addition, we have shown that it is necessary to edit all three copies of *SPO11‐1* to achieve sterility, an observation in contrast to those of Benyahya *et al*. (2020), who showed that, due to a deletion in exon‐2 of the A genome copy of *SPO11‐2*, a double mutation in the B and D genome copies is sufficient to disrupt meiosis and generate sterile plants.

Our results show that triple‐edited, *spo11‐1* wheat lines (*aabbdd*) are completely sterile. This is consistent with results observed in *spo11‐1* silenced rice (Yu *et al*., 2010) and in maize *spo11‐1* mutants (Ku *et al*., 2020). In contrast, *spo11‐1 Arabidopsis* mutants exhibited severely reduced seed set (0.4%–4.4% of wild‐type seed number) but not complete sterility (Grelon *et al*., 2001; Puizina *et al*., 2004; Stacey *et al*., 2006). Fayos *et al*. (2020) have suggested that, given the small number of chromosomes in *Arabidopsis* (*n* = 5), balanced segregation may occur by chance; such serendipity would be highly unlikely in wheat which has 42 chromosomes. However, hexaploid wheat, having three genomes, is more able to tolerate unbalanced chromosome numbers (aneuploidy) than *Arabidopsis*, thus allowing some degree of fertility (Winfield *et al*., 1995; Zhang *et al*., 2013). Interestingly, all our sterile wheat plants, irrespective of their precise genotype, showed varying degrees of distorted ear morphology, usually in the form of extended secondary branches carrying the florets (Figure 4). This phenotype only occurred in sterile lines; all other lines, irrespective of their level of fertility, had normal ear morphology. This might suggest that a failure in the meiotic pathway can lead to changes in the ear developmental pathway resulting in an overproduction of florets.

The sterile phenotype of *aabbdd* plants is explained by the disruption of meiosis observed in these plants. Meiotic recombination is accompanied by extensive reorganization of the chromosomes and the two processes are intimately linked and coordinated (Kleckner, 2006). Part of this procedure involves the close alignment and tight linkage of homologous chromosomes along their length by the SC. Chromosome axis development in the early stages of meiosis appeared cytologically normal in *aabbdd*, but as prophase I progressed chromosomes failed to synapse and the SC transverse filament protein, ZYP1, formed numerous axis‐associated foci rather than polymerizing to give a linear signal. Defective synapsis is a characteristic feature of *spo11‐1* mutants in other plant species (Fayos *et al*., 2020; Grelon *et al*., 2001; Ku *et al*., 2020; Yu *et al*., 2010) and in *spo11* mutants of budding yeast and mouse (Giroux *et al*., 1989; Romanienko and Camerini‐Otero, 2000). In late prophase I, we occasionally observed a few short stretches of linear ZYP1 signal in *aabbdd*. Similarly, limited stretches of ZYP1 signal have been observed in *spo11* mutants of other organisms, including plants, and have been suggested to result from non‐homologous synapsis (Ku *et al*., 2020).

In most previous analyses of plant species where immunocytochemistry has been used to indirectly estimate meiotic DSBs, mutation of either *SPO11‐1*, *SPO11‐2*, or *MTOPVIB* resulted in a dramatic reduction in foci numbers implying a corresponding reduction in DSBs (Benyahya *et al*., 2020; de Muyt *et al*., 2007; Fayos *et al*., 2020; Ku *et al*., 2020; Li *et al*., 2022; Sanchez‐Moran *et al*., 2007; Steckenborn *et al*., 2022). For example, in maize and rice, mutation of *SPO11‐1* was reported to result in the reduction of DSBs by ~97% and ~83%, respectively (Fayos *et al*., 2020; Ku *et al*., 2020). Similarly, in maize and wheat, mutation of *SPO11‐2* eliminated more than 90% of DMC1 foci indicative of a failure to form DSBs (Benyahya *et al*., 2020; Li *et al*., 2022). The reduction of DMC1 foci by only 30% in the triple‐edited *spo11‐1* mutant was therefore initially surprising. Nevertheless, this reduction was statistically significant and, on close inspection, foci in the mutant appeared morphologically abnormal, exhibiting greater heterogeneity in size and shape than in the wild type. This, together with a failure of chromosome synapsis and CO formation is consistent with a defect in normal DSB formation. Notably, a remarkably similar phenotype was described for a rice *spo11‐2* mutant containing a 1906 bp deletion (Fayos *et al*., 2020). This revealed that despite the sterile phenotype, failure to form chiasmata, and lack of chromosome synapsis, the line still exhibited 74% of the wild‐type level of DMC1 foci. Clearly, further studies will be required to explain the residual DMC1 foci in these mutants and whether, at least in the case of wheat, the apparent upstream recruitment of an inactive form of the *SPO11‐1* protein to the chromatin, is implicated in this.

In addition to generating genetic variation, COs provide the physical links between homologous chromosomes necessary for accurate segregation at the first meiotic division. By metaphase I, it was clear that our *aabbdd* plants had failed to form any Cos, with chromosomes presenting as 42 univalents rather than 21 bivalents. Subsequent mis‐segregation leading to unbalanced, inviable gametes would explain the sterility of this mutant. The failure of *aabbdd* plants to synapse or form Cos is entirely consistent with the *spo11‐1* phenotype described in other plants (Fayos *et al*., 2020; Grelon *et al*., 2001; Ku *et al*., 2020; Stacey *et al*., 2006; Yu *et al*., 2010).

Interestingly, the presence of a single copy of *SPO11‐1* was sufficient to maintain synapsis, CO formation, and thus balanced segregation of chromosomes. However, the *SPO11‐1A* homoeologue differed from the other two homoeologues in its synapsis dynamics and its ability to form CO/chiasmata. Several mechanisms exist to control the number and distribution of COs along chromosomes such that plant chromosomes usually contain 1–3 COs and all chromosomes receive at least one, ‘obligate’ CO (Jones and Franklin, 2006; Martini *et al*., 2006). In this study, at metaphase I, a very small number of achiasmate (univalent) chromosomes were observed in wild type, *aaBbdd* and *aabbDd* plants. In contrast, in plants with a single wild‐type copy of *SPO11‐1A* (*Aabbdd*) 45% of nuclei exhibited univalent pairs at metaphase I. Indeed, many had two univalent pairs, a situation not seen in wild type, *aaBbdd* or *aabb*D*d*. Since achiasmate chromosomes often fail to orientate properly on the spindle they are at risk of mis‐segregation at the division stages (Zamariola *et al*., 2014). Although we saw examples of unattached ‘lagging’ chromosomes in *Aabbdd*, most nuclei segregated with the normal 21:21 ratio (85%, *n* = 20), consistent with the near wild‐type level of fertility observed in this line. This is possible because, when there are no more than one or two achiasmate chromosomes per nucleus, they often end up in the correct daughter nuclei by chance.

In land plants, the *SPO11‐1* function appears to be highly conserved. Certainly, *SPO11‐1* coding sequences are highly conserved between species, especially with respect to the seven functional domains (Sprink and Hartung, 2014), and *SPO11‐1* cDNAs have been shown to complement *spo11‐1* mutants in cross‐species transformations (Da Ines *et al*., 2020). Interestingly, full‐length genes were not able to complement due to complex alternative splicing patterns (Sprink and Hartung, 2021). Our results show that *SPO11‐1* mutants have similar phenotypes between species and confirm that in wheat, as in other plants, two *SPO11* orthologues are required for recombination. Evidence from *Arabidopsis* suggests that SPO11‐1 and SPO11‐2 form a heterodimer which, together with two molecules of MTOPVIB, comprises the DSB catalytic complex (Vrielynck *et al*., 2016). The catalytically active tyrosine residues of both *SPO11‐1* and *SPO11‐2* are required for break formation (Hartung *et al*., 2007). Given the high degree of functional and sequence conservation of plant *SPO11* proteins, it seems likely that wheat *SPO11‐1* and *SPO11‐2* similarly form a catalytic heterodimer within a heterotetrameric DSB complex, especially as the MTOPVIB protein is also functionally conserved in cereals (Jing *et al*., 2020; Steckenborn *et al*., 2022; Xue *et al*., 2016).

As our long‐term interests are in modifying recombination in hexaploid wheat, we chose to complement our mutant line with a full‐length copy (8.55 kb) of *SPO11‐1B*. This full‐length copy included ~4 kb of upstream regulatory sequences. We chose to include such a large segment of upstream sequence because our previous, unpublished work, using *SPO11‐1* promoters of ~1 kb failed to show any evidence of mutant complementation. In this respect, it is interesting to note that in a diverse range of plant species from *Arabidopsis* to maize, including wheat, the *SPO11‐1* upstream regions overlap with another gene. It is interesting to speculate on why these two genes have remained in tandem throughout at least 60 million years of evolutionary history and if such an alignment is important for *SPO11‐1* regulation. To reduce the time required to grow and cross wheat, we directly transformed the triple mutant line, a procedure that, to the best of our knowledge, has not been previously reported. We estimate this approach saves up to 12 months on the standard procedure of transforming wild‐type plants for further crossing. Complementation of *spo11‐1* lines with a full‐length *SPO11‐1B* was able to restore CO formation, synapsis, and fertility (Figure 8) although the level of fertility restored appeared to be dependent on the level of transgene expression, with higher levels of transgene expression leading to increased seed set. To date, these transgenic plants have maintained their levels of fertility to T_4_ generation and as such they are a valuable resource for studying the effect that different levels of *SPO11‐1* protein have on meiotic progression, CO frequency, and fertility.

In future studies, we aim to modify the DSB landscape either by selective targeting of DSBs via, for instance, the inclusion of targeting sequences such as zinc fingers or by influencing the timing of DSB formation in different chromosomal regions and thereby modifying CO distribution. Here, we have reported the development of gene‐edited lines and functional *SPO11‐1* constructs that are a prerequisite to achieving these longer‐term goals.

### Experimental procedures

#### Plant and growth conditions

Wheat cultivar Cadenza and the transgenic derivatives were grown in 5‐inch pots containing Sinclair, peat‐based compost (Sinclair, Cheshire, UK). Plants for transformation were grown in a controlled environment chamber at 18/14 °C day/night and 16/8 h light/dark cycle, whilst plants grown for general purposes were grown in a greenhouse under the same conditions.

#### Plasmid construction and transformation

Plasmid Cas9.P2A:H2A:GFP carrying a maize ubiquitin promoter controlled wheat‐optimized Cas9 along with two guide RNAs and a GFP reporter construct (on a single plasmid) was generated as described in Zhang *et al*. (2019). Before carrying out experiments designed to generate transgenic wheat lines, all constructs, including guide RNAs 4 and 6 specific for all three homoeologues of *SPO11‐1*, were tested in wheat protoplasts as described by Zhang *et al*. (2019). To generate transformed Cadenza lines, immature embryos were subjected to particle bombardment as previously described by Sparks and Doherty (2020). Plants carrying edit events were identified via PCR using generic *Spo11‐1* primers (File S2). Amplification was carried out for 35 cycles using the following conditions: 94 °C 30 s, 58 °C 30 s 65 °C 3 m, followed by Sanger sequencing of PCR products.

For the transformation of *spo11‐1* edited lines, immature embryos were isolated from *spo11‐1 aaBbdd* lines (i.e., embryos segregating for wild‐type and mutant *spo11‐1 5B*) and co‐bombarded with *pSPO11‐1::SPO11‐1‐5B* containing the *SPO11‐1* promoter, *SPO11‐1‐5B*, and the *SPO11‐1* terminator, and *pUbi::Hyg* containing the hygromycin resistance gene. Once transferred to soil, *SPO11‐1* triple edited lines (*aabbdd*) containing *pSPO11‐1::SPO11‐1‐5B* were identified via PCR using B genome‐specific *spo11‐1* primers (File S2). Amplification was carried out for 35 cycles using the following conditions: 94 °C 30 s, 58 °C 30 s 65 °C 3 m.

#### Cytological procedures

Chromosome spreads were prepared from anthers at the appropriate stage of meiosis. For chiasma visualization, metaphase I anthers were fixed and stored in cold ethanol:acetic acid (3:1 v/v) and slides prepared as described in Howell and Armstrong (2013) with the following changes: up to 3 anthers per slide were macerated in 1 μL water, 2 × 10 μL of 70% acetic acid was added and the slide incubated on a 45 °C hot‐plate for 90 s with constant stirring then fixed with 130 μL cold ethanol:acetic acid (3:1 v/v) and DNA was stained with 5 μg ml^−1^ 4′,6′‐diamidino‐2‐phenylindole (DAPI) in Vectashield (Vector Labs).

For immunolocalization, slides were prepared from prophase I anthers based on a method previously used for *Brassica oleracea* (Armstrong *et al*., 2002) with the following modifications: ~20 anthers were digested in 20 μL enzyme mix (0.4% cytohelicase, 1.5% sucrose, and 1% polyvinylpyrrolidone) in a cavity slide inside a humidified box on a 37 °C hot plate; after 4 min, anthers were gently crushed to release PMCs, debris was quickly removed with a needle and digestion continued for a further 3 min. The PMC suspension was then used to prepare up to 4 slides by transferring 2 μL aliquots to 10 μL of 1% lipsol (in water) on slides, gently spreading with the side of a needle and fixing in 12 μL fresh, cold 4% paraformaldehyde solution. Subsequent immunolocalization steps were carried out as described in Armstrong *et al*. (2002). Primary antibodies were used at the following dilutions: anti‐*At*ASY1 rabbit or guinea pig, 1:500 (Armstrong *et al*., 2002); anti‐*At*ZYP1 rabbit or guinea pig, 1:500 (Higgins *et al*., 2005; Osman *et al*., 2018); anti‐*At*SPO11‐1 rabbit, 1:200; and anti‐DMC1 rabbit, 1:200 (Sanchez‐Moran *et al*., 2007). Secondary antibodies were FITC (green), Alexa Fluor 350 or AMCA (blue), or Alexa Fluor 594 (red) conjugates used according to the manufacturers' instructions (Sigma; Thermo‐Fisher; Jacksons ImmunoResearch).

Images were captured as Z‐stacks with a 0.2 μM z‐step using a Nikon Eclipse 90i fluorescence microscope fitted with a Nikon DS‐Qi1Mc camera and NIS Elements capture software. Chiasmata and immunolocalization foci were counted manually by examining all individual z‐frames using the raw data files; the count tool in NIS Elements was used to mark scored foci to avoid double counting when moving between frames. Differences in chiasmata and foci frequency were tested for significance using single‐factor ANOVA.

## Author contributions

K.O., M.W., E.S‐M., J.D.H., I.R.H., F.C.H.F., and K.J.E. designed the research. L.H., K.O., C.S., and K.J.E. performed the experiments. L.H., K.O., M.W., E.S‐M, J.H., I.H., F.C.H.F., and K.J.E. analysed and interpreted the molecular data. K.O., ESM and F.C.H.F. analysed and interpreted the cytological data. L.H., K.O., and M.W. wrote the manuscript.

## Conflict of interest

The authors declare that there is no conflict of interest with regard to the work carried out for this study or in the contents of the manuscript.

## Supporting information


**Figure S1** Sequences of TaSpo11‐1A/1B/1D from Chinese Spring as downloaded from the Ensemble Plants web site (https://plants.ensembl.org/Triticum_aestivum/Info/Index). In each case exons are in red font and introns in black font, the 3' UTR is highlighted in grey; the location of the guide RNAs are highlighted in yellow (sgRNA 6 in exon 3 and sgRNA 4 in exon 4); the codon for the active site tyrosine is highlighted in sky blue. Sequence comparison between Chinese Spring and Cadenza revealed that there were no sequence variations in the region covered by both the active site and the two guide RNAs.
**Figure S2** Confirmation of *Spo11‐1* sgRNAs activity in wheat protoplasts. Wheat protoplasts were transformed with (i) just the wheat optimised Cas9 construct (described by Zhang *et al*., 2019) or (ii) with the Cas9 construct plus the *SPO11‐1* specific guide RNAs sg4 (5′GCACAGACCTACGAACATGG3′) and sg6 (5′GGAGAGGACGTCCGTGCCGA3′). (a) High efficiency of wheat protoplast transformation as evidenced by a nuclear targeted, GFP reporter. (b) *Spo11‐1* specific guide RNAs were designed to generate a deletion of ~200 bp following amplification with primers designed to flank the region. Fragments were electrophoresed on a 2% agarose gel: (SS) New England Biolabs 1kb ladder; lane 1 is the negative control; lanes 2, 3 and 4 are from protoplasts transformed with both guide RNAs. (c) Aligned sequences of the larger (c. 500 bp) and smaller (c. 200 bp) bands after excision and sequencing; the sequences of the two guide RNAs are also shown.
**Table S1** Expected and observed genotypes of T1 progeny from plant 22

**File S2** PCR Primers.
