# Supplementary material for: Identification, characterization, and rescue of CRISPR/Cas9 generated wheat SPO11‐1 mutants
Source: Plant Biotechnol J. 2022 Dec 10;21(2):405–18. doi: 10.1111/pbi.13961 (PMC9884015; doi:10.1111/pbi.13961)
Supplement: Supplementary file 1 — Figure S1 Sequences of TaSpo11‐1A/1B/1D from Chinese Spring as downloaded from the Ensemble Plants web site (https://plants.ensembl.org/Triticum_aestivum/Info/Index). In each case exons are in red font and introns in black font, the 3' UTR is highlighted in grey; the location of the guide RNAs are highlighted in yellow (sgRNA 6 in exon 3 and sgRNA 4 in exon 4); the codon for the active site tyrosine is highlighted in sky blue. Sequence comparison between Chinese Spring and Cadenza revealed that there were no sequence variations in the region covered by both the active site and the two guide RNAs. Figure S2 Confirmation of Spo11‐1 sgRNAs activity in wheat protoplasts. Wheat protoplasts were transformed with (i) just the wheat optimised Cas9 construct (described by Zhang et al., 2019) or (ii) with the Cas9 construct plus the SPO11‐1 specific guide RNAs sg4 (5′GCACAGACCTACGAACATGG3′) and sg6 (5′GGAGAGGACGTCCGTGCCGA3′). (a) High efficiency of wheat protoplast transformation as evidenced by a nuclear targeted, GFP reporter. (b) Spo11‐1 specific guide RNAs were designed to generate a deletion of ~200 bp following amplification with primers designed to flank the region. Fragments were electrophoresed on a 2% agarose gel: (SS) New England Biolabs 1kb ladder; lane 1 is the negative control; lanes 2, 3 and 4 are from protoplasts transformed with both guide RNAs. (c) Aligned sequences of the larger (c. 500 bp) and smaller (c. 200 bp) bands after excision and sequencing; the sequences of the two guide RNAs are also shown. Table S1 Expected and observed genotypes of T1 progeny from plant 22 [file PBI-21-405-s002.docx]

**Figure S1.** Sequences of TaSpo11-1A/1B/1D from Chinese Spring as downloaded from the Ensemble Plants web site (<https://plants.ensembl.org/Triticum_aestivum/Info/Index>). In each case exons are in red font and introns in black font, the 3' UTR is highlighted in grey; the location of the guide RNAs are highlighted in yellow (sgRNA 6 in exon 3 and sgRNA 4 in exon 4); the codon for the active site tyrosine is highlighted in sky blue. Sequence comparison between Chinese Spring and Cadenza revealed that there were no sequence variations in the region covered by both the active site and the two guide RNAs.

***TaSpo11-1A* from Chinese Spring (TraesCS5A02G391400)**

>5A dna:chromosome chromosome:IWGSC:5A:586599336:586604800:1

CCGAGAAGCGGAGAGCTCGTCGTTCATCGTTCTGGTAGAGTTCCCGGTGGCGCAGCTCAA

TAGCAAGAGGTCCAGGCCTTCGACGAGCAAGAGTAACTTCTGAATCCCGACGACGGGTGT

CCTCGAGGTGTGAACCAGGGCTATTGATTGGGTTGCGCAAAGAGCAAGCCCTTTGGGAGA

GCCTAGCTGATGTATCATATGTGTAGCTGGGAACATGGGTTGTTCCCATCTTTGATGGTT

GAGGAGGAGCTTAGCTAGCTTAGCTGTTGTTAGGTTTTTAGTGTAATTTTAACACGGCAA

CTCTACGAGTGCGCGATGAAATAAAATTAAAATATGTGCTTGTGTTCTTCGATCAATTCT

CAGGTGGTTGCGAATTCAGACGTTCAGTCTGATTTGAGATTCTTGTTCTTCTGTATTTTC

AGATACTGCAGTGCAGTTTGCAGAGTATACTACACTTGTGTGAAGAAAAAAATAAATAAA

CCATGCGTTTTAAACAGGCCTTGTAGCAGAGGAGGCTTTCACAAAGCCCATTAGACACTA

GCCGTTGCTGGACCAGTTGTGACTTGTGACGGCGCTCTCCTGCTCTCCGAGCGACCGGTC

ATG**GCGGGGAGTGGGAAGAGGAGGCGCGCGACGGTGCTCGACGACGACGAGCGGCGGGGG**

**CGGCGGAGGCTGGAGGAGGCGGCCCTGCTCCTCCACAAGATCAAAG**GTGCGAATCCCCTT

CCCTCTCCTCGATCGACTGTAAACCCTCGTAATCCCCTTCGAATTGGGGTTGCAATCGGA

GTTAACGCGGGTTTGCGCCGCCGCGCGCGTGGGCTGTCGGCGCAG**GGCTGG**T**GGGCTGGG**

**TGGTCGCGGAGGTCAGCGCCGGCCGATCCCCTTCCCTGGCTCTCCACCGCTACCAGAACT**

**ACTGCGCCTGCGCCTCCGCCGCCGCCGCCGCCGCGTCCCTGTCCACATG**GTTTGGCTCCC

CCTCCCTCTCTCGCCTTCAAATCTCTGCACACCAAACCTGTCAGATCCTTACCGGTGTGT

GCGCGTCCGCGGGGTGCGGGCGCGCGTGCAG**CGCCTGCAGCTACGACGCCCCCGTCGGCA**

**CGGACGTCCTCTCCCTCCTCCGCAAGGAATTCCACGCCTCCCGACTCA**GTACGGACTCGC

GTAGACCAAACTGGCTCGATTCTCATCAATCTCTCGTTGTTACTGGTCATGCTAATCAGT

TTGGGATGCGTCGTTGATGTGCAG**ATGTGCTTCTCAGGGTCCTGCTCGTGGTGCAGCAAC**

**TCCTGCAGGAGAATAAGCACTGCTCCAAGAGGGACATCTACTACATGTACCCCTCCATGT**

**TCGTAG**GTCTGTGCTGGTCTGCGGTTGCCATGTCTGATCCATCTATGCGTAGTGTGAGTT

TCAGGTGTTCTCTTTCTTTGCTGTGTGAGTGACAATTACAAGTCTCACTGCAG**AACAAGC**

**GATTGTTGATCGTGCCATCAATGATATCTGCATACTCTTCAAGTGCAGCCGGCATAATCT**

**CAACGTG**GTATGTGCTACCGCTTGTCTGCCATCTTTGCAGAAGCTTCAGTTATGTGTTTA

GAAGTCATTTGTGCATTGGAGAATAAGCCTCTGGCTGTTATTAGATTATATATGGAGTAC

AATGTGCCGCACTGCGACTTGTTACTCCCTGGTTGTTCCTCCATTATGGTGTACCACTGT

CCTATCTGATGTAGGATTATTTTCTTAATCTGTCTTTTGTGATGCATTATTTCTTGAGGT

CTTCATTTCTCCATGAATCGCTAGGAGCTGTTTTCATTAGTTCTCTCAGTGATTTCTGCT

GTATTTATTTCACCGCCTTTTCACTTCTCTTTTAATAACCACCTGCAATTTCTGCTCACT

TGGCTGCTTGCAG**GTTCCTGTGGCAAAAGG**GTATGGATTCTTATTGAGTTGTGAGTGTTT

GATTTTGATAACATGTTTAATCCTTCTATCTTGTCCCATGCCGTTACTTAACTAATCAGC

ATTTTTGTTCCTTCATCCTCCCTACCACTTGTCTTGCTTTACTTAAAACATGATCTGGCT

AAGCAGTGTGATGCTGCGCTTTTAGCAGACATTTAGAGCATCATAATCTCCTGTGTCCAT

ACGATGGTCTTAGTTATTTCTGAACATTTTCCCGCTATGTACTCGTCAATATCTTTATTG

TTCCAG**TTTGGTGATGGGCTGGATAAGATTTGTGGAGGGTGAAAAGAAAGTGTATTGTAT**

**AACAAACGTCAATGCT**GTAAGCTTCTATGATTCTATCGAAATCTATCTAATTACTCAATA

TGATCTGTTTGTACTATGCATTAATTTGGCTAGCCTTTTTCCTTCTTCAAATGCAG**GCTT**

**TCCCCATTCCAGTTAGCATTGAGGCAATCAAAG**GTTGTTTAACAATTTGAAAACTCATAA

TAATCTTTGCCATTCATAGACTTTTATTATTTCATATCTGTTTCCTTAGGAAAATCAGTT

TTAACATCTCTTAGCAAGTCAATGTTCAGTTTAAATTTGTAATAGATAGTGATTGATGTA

TCAGAAAGTGGGATTTCGACAAAGCACCAAATTTTGGACATCATGTGAAAGAGTAATGAG

AATTCATCTTTATTTTTTGTCTCAAACATATATGGAGAGGAGTATCCTTGTTATTCCACA

AACTATATGGCATAGGTTTGCTGTCTTTTTATACATACACTATATTTCTTTCATATTCTC

AATTACTATGCAGTAAACTTAAGCTGTCTAAGTTTTTTCTTGCAG**ATGTTGTTAGTGTTG**

**CTCACTACATCCTTGTTGTTGAGAAGGAGGCAG**GTGTGAGGATTAAAGACCCCCTATCTG

CATATGATTTTTCTATTCCTTGCAGTCTTCCTGCAGTTTGATTCTGTATGCCATTTGCAG

**TGTTCCAGCGTTTGGCCAATGACAAGTTCTGCGAAAAGAATCGCTGCATTGTTATTACA**G

TAACTGCTAACGCTGCATCTAGTGTTATACTGCCATTATTTTTCACATAGCATGTTTGAT

GGTTTTCTTCTGTTTACTCAG**GGAAGAGGCTACCCAGATATTCCAACAAGAAG**GCATGTG

CTCTTTCGTATATTTGTTTCTCCTGTATATTAAAACTGAATTGCTATTTGAAACGTACAT

TCCTTGTAATGTTGCAG**ATTCTTGCGCTACCTTGTCGAACAGCTGCACTTGCCTGCTTAT**

**TGCTTAGTGGATTCAGATCCGTATGGTTTTGATATTCTGGCTACCTATAAATTTGGTTCC**

**ATG**GTAGGGCCAAAATATCTATCTAATATCTAGTTATTTATTGAAGTCCTAATAGCATCA

AAGGCAGTGTAAATTTATCAGTGCAATTTCAATTTTTTTGTTAAGCCAAGTGAAATGTGA

TTTTGTACATTTATTCAAAATTTAGTCCCATTTTGTTGATATGTTTCGAGTGTTAACTGA

CAAACACTTCCTGCTCCCTTGTCCAATTCTCATTCTATTTAAG**CAATTGGCATATGATGC**

**AAATGTTCTGCGTGTGCCTGAGATACGATGGCTTGGGGTCTTCACATCTGACTTTGAGGA**

**GTATTGTCTTCCAGACTGCTGTCAGCTTCAATTGTCATCTGAAG**GTCACTAAATTGGAGG

GATATAAATTTCATTTCTACTTATCTTGGAACTTGATTTGTTGTTTAATATCAAATGACA

TGCATCCTGCTTCAG**ATAGGAGGAAGCTTGAAGGAATTCTAACCAGATGTTACTTGCACA**

**AGGAAGCCCCAGAATGGAG**GTAAAGGAACAGGAAGTTGTATTTCCTATTACACGTATTCC

AATAACTTTATGGCTATGATGTGGGTTGCAAGAATAGTTAGTGCATCTAGAATTAAATAG

AAGTTCTCACAATATCTTTCTTACAAAATGGCAAATGCAACAGTTAAACTCAGTGATGTA

AGTGAAATCTTCTTCCAAGCACAATGATCAATCCTATTATTTAGCCCCTGTATTCCAAGC

ACTTCAGGGATATGATGTGGGTTGCAAATAATAATAGTTCAAGTCCAACTACAAGAATTT

GTTATTTGATTTGTTTAATCAACTAGTAGTTAAATAGAGCTTTTCATATTTTCTTCTTAC

AAAATGCCAAATGCAACAACAAAATTCACTGATACGAGTGAAACCTTCTTCCAGTACACA

GTCTTATGATTTTATCCCGTGTCCGTAATATGTAAGTGCTTTCAGCATCAGTTCCTTAGT

TTCAAAAACTCCATATCTGCGCCACCTTGTCGACTCTAACAGCAGTAGGTGCTCCATTGT

TCCAAAAGGTCTATGACTGCTTTCACCTGGTCAGTTCTAATGTCAACGGGTTTGTGTTTA

AAATTAGAATCATTTATGTAGTTAATAGCCGGTTCTTTTTCCTGAAGATTTTTCCTCCTG

AAATATTCACGACTATATAATCCTTGGCAG**GTTGAAGTTGGAAGCCATGCTGGAAATGGG**

**TGTCAAGTTTGAGATTGAAGCATTGTCAGCAAGTTCCATTTCGTTTCTGTCGCAAGAGTA**

**CATCCCCCAACAGATCAGGCTAGGGAGGTATATATAG**GATTGCATAGACCTGTATCTATG

CAGAATTGTGCTAGGCTAAGAGTCTCCTTTTGTTTTGGTTACACCACTTGTAGTTGCTTG

TCTGATCATACGTCAAACAATCACCTTGGTATTCACAACAAGAGTTTGACTGCAATTGCT

GTATACCTTCCATTACTGCATTGCTACTCATAATGCTTCTTGTGGTGCGTAGATTAGTTT

GGGTTTATTGTGTTATTAATGTGTAAAGGCAACCTGTTAGAAGTGAATGCCTGAATAGAA

ACTCTGGATGTCCTCTTAGTTCTACCAGGTCACCATCTCATGGAAGCAAAATCATATTTA

CCTGAGATCTCAGCCTCCACTTGCCGAGCATCATTAGATAGGTTGTTGCTTGCCTTGCAA

TTCTGGAGCCAGGAGAAACAAATCCAAGAAATACATCTACACAGGCTACCATATTGGAAG

CTGGAAATATTTCATGGCTGGAAAATAGTTTAACAGACTTCATGTACACAGGTACTTAAG

CTTAGCGAAGCATCACCAAATACTTCTTTCTTACACATGTCCAGGTGCATATGAGCAGAT

CCATTGTGTTAAATACATGTAGGAGCTCAACGAGGTCGTATCTTTTAACACTTTACTAGA

TTACAGAATTAATTAACAGCATTAACCGACTATTCAACCAAAGTATTTAAGCTGTAGAAT

TAGGTACAAGCCCATATGTTCGTCACTGACCGGATTGTGCCTGAGTAAGGTCATATGTGG

AGAGCGTGCCGGACTGGTCAACGTCGAGTTTGTCAAACTCCTCTAGAAAAGAAGATATGT

CCTCTTGGCTGATCTTCCCCAGTTCTTTGAGCTTGTACACGACAAATTCAGCAGCACTGC

ACATATCAAAGTTCACAAAATCAACATCAATTGCTCATTACCTGCAAAATCTCAAGGGAT

ATGAT

***TaSpo11-1B* from Chinese Spring (TraesCS5B02G396300)**

>5B dna:chromosome chromosome:IWGSC:5B:573221084:573228916:1

CTCGTCGTTCATCGTTCTGGTAGAGTTCCCGGTGGCGCAGCTCAATAGCAAGAGGTCCAG

GCCTTCGACGAGCAAGAGTAACTTCTGAATCCCGGCGACGGTGCCCTCGAGATGTGAACC

AGGGCCATTGATTGGGTTGCTGGAAGAGCAAGCCCTTTGGGAGAGCTTAGCTGATGTATC

ATATGTGTAGCTGGGAACATGGATTGTTCCCATGTTGATGGTTGAGGAGGAGCTTAGCTA

GCTTAGCTGCTGTTAGGTTTTTAGTGTAATTTTAACACGGCAACTCTACGAGTGATCGAT

GAATAAAATTAAAATATGTGCTTGTGTTTTTTGATCAATTCTCGGGTGGTTGCAAATCCA

GACGTTCAGTCTGATTTGAGATTCTTGATCTCCTCTTTTTTCAGTTACTGCAGTGCAGAG

TACACTACTATTTTATTTAGGCTTGCAGAGTACACTACACTACAATTGTGAAGAGGAAAA

AAAACCGTTCTAAACAGGCCTTGGAGCAGAGGAAGCTTTCACAAAGCCCATTAGACACTA

GCCGTTGCTGGACCAGTTGTGGCTTGTGACGGCGCTCTCCTGCTCTCCGAGCGACCGGTC

**ATGGCGGGGAGTGGGAAGAGGAGGCGCGCGACGGTGCTCGACGACGACGAGCGGCGGGGC**

**CGGCGGAGGCTGGAGGAGGCGGCCCTGCTCCTCCACAAGATCAAAG**GTGCAGATCCCCCT

TCCCTCTCCTCGATCGACTGTAAACCCTCGTACTCCCCTTCGAATTGGGGTTGCAATCGG

AGTTAACGCGGGTTCGCGCCGCCGCGCGCGTGGGCTGTCGGAGCAG**GGCTGGTGGGCTGG**

**GTGGTCGCGGAGATCAGCGCCGGCCGATCCCCTTCCCTGGCGCTCCACCGCTACCAGAAC**

**TACTGCGCCTGCGCCTCCGCCGCCGCCGCGGCGTCCCCGTCCACATG**GTTTGGCTCCCTC

TTCCCCACTCTCTCGCCTTCAAAATCTCTGCCGACCAAGCCTGTCAGATCCTCACCGGCG

TGTGCGCGTTCGCGGGGTGCGGGCGCGCGTGCAG**CGCCTGCAGCTACGACGCCCCCGTCG**

**GCACGGACGTCCTCTCCCTCCTCCGCAAGGAATTCCACGCCTCCCGCCTCA**GTACGGATT

CGCGTACCCCAAACTGGCTCGGTTCTCATCGATCTCTCGTTGTTTCTGATCATGCTAATC

AGTTTGGGATGCGTCGTTGATGTGCAG**ATGTGCTTCTCAGGGTCCTGCTCGTGGTGCAGC**

**AGCTCCTGCAGGAGAATAAGCACTGCTCCAAGAGGGACATCTACTACATGTACCCCTCCA**

**TGTTCGTAG**GTCTGTGCTGCTCTGCGGTTGCGATGCCTGATCCATCTATGCGTAGTGTGA

GTTTCAGGTGTTCTGTTTCTTTGCTGTGTGAGTGACAATTACAAGTCTCACTGCAG**AACA**

**AGCGATTGTTGATCGTGCGATCAACGATATCTGCATACTCTTCAAGTGCAGCCGGCATAA**

**TCTCAACGTG**GTATGTGCTACCGCTTGTCTGCCGTCTTTGCAGAAGCTTCAGTTATGTGT

TTAGAAGTCATTTGTGCATTGGAGAATAAGCCTCTGGCTGTTATTTCATTATGGTGTAGT

GGTGTACACAATGTGCCACACTGCCACTTGTTACTCCCTGGTTGTTCTCTGTCCTATCTG

ATGTAGGAGCATTTTCTTAATCTGTCTTTTGTGATGCATTATTCATTGAGGTCCTCATTT

CTTCATGAATCGTCAGGAGCTGTTTTCATTAGTTCTCTCAGCAGTTCTGCTGCATTTATT

TCATCGCCTTTTCACTTCGCTTTTAGTAACCATCTACAATTTCTGCTCACTTGGCTGCTT

GCAG**GTTCCTGTGGCAAAAGG**GTATGGATTCCTATTGCGTTGTGAGTGTTTAATTTTGAT

AACATGTTTAATCCTTCGATGCTGTTACTTAACTAATCAGCATTTTTGTTCCTTCATCCT

CCCTACCGCTTGTCCTGCTTTACTTAAAAAATGATCTGGGTGTAAACAGTGGGATGCTGC

TCTTTTAGCAGAGATTTAGAGCATCACAATCTCCTATGTCCATACAATGGTCTTAGTTAT

TTCCGAACATTTTCCCGCTGTGTACTCGTCATGTCTTTATTGTTCCAG**TTTGGTGATGGG**

**CTGGATAAGATTTGTGGAGGGTGAAAAGAAAGTGTACTGCATAACAAACGTCAATACT**GT

AAGCTTCTATGATTCCATCTAATCACCCAGTATGATATGTTTCTAGTATGCATTAATTTT

GCTAGCCTTTTTCCTTCTTCAAATGCAG**GCTTTCCCCATTCCAGTTAGCATTGAAGCAAT**

**CAAAG**GTTGTTTAACAATTTGAAAACTCATAACAATCTTTGTCTTCATAGTTTTTTATTA

TTTCATATCTGTTTCCTTAGGAAAAACAGTTTTATAAATCGTGATGTACACAATTAGCTT

ATATCTCTTAGCAAGTCAATGTTCAGTTTAAATTTGTAATAGATAGTGATTGATGTATTA

GAAAGTGGGATTTCGACAAACAACCAAATTTTGGACAGCATGTGAAAGAGTAATGAGGAT

TCATCTTTATTTTTTGTCTCAAACAAATATGGAGAAGAGTATCCTTGTTATTCCACAAAC

TGTATGGCATAGGTTTGCTGTCTTGTTATATGTACACTATATTTCTTTCATATTCTCAAT

TACTATGCAGTAAACTTAAGCTGTCTAAGTTTTTTCTTGCAG**ATGTTGTTAGTGTTGCTC**

**ACTACATCCTTGTTGTTGAGAAGGAGGCAG**GTGTGAGGATGAAAGGCCCCCTGTCTGCAT

ATGATTTTTCTATTCTTTGCAGTCTTCCTACAGTTTTCTTCTGTATGCCATTTGCAG**TAT**

**TCCAGCGTTTGGCCAATGACAAGTTCTGCGAAAAGAATCGCTGCATTGTTATTACA**GTAA

CTGCTAACGCTGCATCTAGTGTTATACTGCCATTATTTTTCACATAGCATGTTTGATGGT

TTTCTTCTGTTTACTCAG**GGAAGAGGCTACCCAGATATTCCAACAAGAAG**GCATGTGCTC

TTTCATATATTTGTTTCTCTTGTATATTAGAACTGAATTGCTATTTGAAATGTACATTCC

TTGTAATGTTGCAG**ATTCTTGCGCTACCTTGTCGAACAGCTGCACTTGCCTGCTTATTGC**

**TTAGTGGATTCAGATCCGTATGGTTTTGATATTCTGGCCACCTATAAATTTGGTTCCATG**

GTAGGGCCAAAAATCTTATTTATTGAAGTCCTAATAGCATCAAAGGCAGTGTAAAATTAT

CAGTGCAATTTCAAATTTTTTGTTAAGCCAAGTGAAGTGTGATTTTGTACATCTATTCAA

AATTTAGTCCCATTTTGTTGATATGTTTCTAGTGTTAACTGACCAACTCTTCCTGCTTCC

TTGTCCAATTCTCATTCTATTTAAG**CAATTGGCATATGATGCAAATATTCTGCGTGTGCC**

**TGAGATACGATGGCTTGGGGTCTTCACATCTGACTTTGAGGAGTATTGTCTTCCAGACTG**

**CTGTCAACTTCAATTGTCATCTGAAG**GTCACTAAATTGGAGGGATATAAATTTCATTTCT

ACTTCTTATCTCAGAACTTGATTTGTTGTTAAATATCAAATGTGATGTATCCTGCTTCAG

**ATAGGAGGAAGCTTGAAGGAATTCTCACCAGATGTTACTTGCACAAGGAAGCCCCAGAAT**

**GGAG**GTAAAGGAACAGGAAGTTGTATTTCCTATTACGCGTATTCCAATAACTTTGTAGCT

ATGATGTGGGTTGCAAGAATAGTTCAAGTGCATCTAGAATTAAATAGAAGTTTTCACAAT

ATCTTTCTTACAAAATGGCAAATGCAACAGTTAAACTCAGTGATGTAAGTGAAATCTTCT

TCCAAGCACAAGGATCAATCCTATTATTTAGCCCCTGTATTCCAAGCACTTCAGGGATAT

GATGTGGGTTGCAAATAATAATAGTTCAAGTCCAACTCTAGAATTTGTAATTTGATTTGT

TTAATCAACTAGTAGTTAAATAGAGCTTTTCATGTTTTATTCTTACAAAATGCCAAATGC

AACAACAAAACTCACTGATATGAGTGAAATCGTCTTCCAGTACAATTATCAGTCTTATGA

GTTTATCCCGTGTACGTAATCTGTAAGTGCTTTCAGCATCAGTTCCTTAGTTTCAAAAAC

TCCATATCTGCGTCACCTTGCCGACTCTAATGGCAGTAGGTGCTCCACTGTTCCAAAAGG

TTTATGACTGCTTTCACCTGGTCAGTTCTAAAGTCAACGGGTTTGTGTTTAAAATTAGAA

TCATTTGTGTAGTTAATAGCCGGTTCTTTTTCCTGAAGATTTTTCTTCCTGAAATATTCA

TGACTATATAATCCTTGGCAG**GTTGAAGTTGGAAGCCATGCTGGAAATGGGTGTCAAGTT**

**TGAGATTGAAGCATTGTCAGCAAGTTCCATTTCGTTTCTGTCGCAGGAGTACATCCCCCA**

**ACAGATCAGACTAGGGAGGTACATATAG**GATTGCATAGACCTGTATCTATGCAGAATTGT

GCTAGGCTAAGTGTCTCCTTTTGTTTTGGTCACACCACTTGTAGTTGCTTGTCTGATCAA

ACAATCACCTTGGTATTCACAACAAGAATTTGACTGCAATTGGTGTATACCGTCAGTTAC

TGCATTGCTACTCATAATGCTTCTTGTTGTGCATAGATTAGTTTGGGTTTATTATGTTAA

TGTGCAAGGCAACCTGTTAGAAATGAATGCCTGAATAGGGAACTCTGGATGTCCTCTTAG

TTCTACTAGGTCACCATCCCATGGAAGCAAGTTCCTATTTACCTGAGATCTTAGCCTCCA

CTTGCCGAGCATCATTAGATAGATTGTTGCTTGCCTTGCAATTCTGGAGCCAGAAACAAC

AATCCAAGAAAAAAAAATGCATCTACACAGGCTACCATAGTGGAAGCTGGAAATATTTCA

TGGCTGGAAAATAGTTTAACAAACTTCATGTACACAGGTACGTAAGCTTAGCGAAGCATC

ACCAAATACTTCTTTCTTACACATGTCCAGGCGCATATGAGCATATCCATTGTGTTAAAT

ACATGTAGGAGCTCAACAAGGTCGTATCTTTTGACACTTTACTAGATTACAGAATTAATT

AACAGTGTTAATCGACCATTCAACTAAAGTATTTAAGCTGTAAGATTAAGTACAGGCCCA

TATGTTCGTCACTGACCGGATTGTGCCTGAGTAAGGTCATATGTGGAGAGTGTGCCGGAC

TGGTCAACGTCGAGTTTGTCAAACTCCTCTAGAAAAGAAGATATGTCCTCTTGGCTGATC

TTCCCCAGTTCTTTGAGCTTGTACACGACAAATTCAGCAGCACTGCACATATCAAAGTTC

ACAAATCAACACTCATTACCTACAAAATCTTAGGATATGATGGAGGGAAGAATACTGAAA

ACGCATTATGTGCTTACCCCACTTTCCGATCATTATCCAGATCAGCTGCTTCAAGATCCA

TGGTTGTTACTCTCCGTGTGAGAACCCATTTGGCCAGCATCTTTTGCCGTCGCTCGGTGT

AGATCTCGGCGAGGTACATGAAGAAGAGCGCCAGAATTATGGTGCTTGTAATTATCCAAA

ACACCGCGAAAGTGCGCCCCAGTTGGGACGAGAAGCTTTTATCCCCATAACCCAAGGTAG

TGATTGTGGCACAGACACAATAAAAGGAGTCAACAAGGCTCAGCTTCTCAACCTTCCAGA

GAAAAACAGTCCCCACAACAATGGCCGTCACAAGAAGCAGAGCGGCCGTGTAGAACTTGT

ACTTTGTCTTGTTTGTCTCAATTTGTCTGAGCATTCTGGCCTCGCCACACTTCATATTCA

TGTGCAGTGCCTTGAAGAACAACACCTCCTGCTTCTCGACGAGATAGTCTGCCGACTTGC

TCACGAAGAGAGCAATAATCGCCATGCCGGTGAAGACGAAAACACAAGCGAGCAGCTTTG

TCGTGTCGCTGTTAGGAACAAGGTCTCCATAGCCCACCGATGTCATCGTGACAATGCAGA

AGTACAGTGCGTCGAGCACTCTGTTGGTTCTCTTGCCAGATAACTGATCCATGACAAGGT

AAAAGACTACGACGCCAACTAGCAGGTAGAGAAATAGGAGGAACCCTACTAGTCTGAAGC

TGGGCCGTACCACGCTGAACAACTCCTTGGCTGGAAGCGACGAGCCATTTTCTTGTGGTT

TTTCATCGGTCTCTGATCTGGGAGCCGACCTGCATCTTCGGAACCGCTTGGCTCCTTGCG

ACGGCTTTCTTTGGAGCACATTGGCATCGGGGTTCTCAGGTAGCAATGCTCGCTGAATGC

TGTTGTCAGACATTGGCTTATGCTGGATTTGTTGTGAATTGCCGTCGCAATTGAAGCCCT

ATGATTCAGAGAATGGCACACGTTAGAGTATGGTTTTACAGATATGCGAGGAAGAAGTTT

AGAAGCTCATTAAATATCAGGTAAAAAATAAGGCAAGACCGTGGATATGCACTGAATTGC

ATGAACCACCGAGAAACTCTATTCCTCTCAGCTTGTGTGAATTATACGTGTCCTAGCTGA

CACAAGAATTTCCTATCACCATCAGTTTAAGCATTAAATTATTGGACTGCAGGTCAAAGA

GAGGCAGGCAAAATAAGTTTGGTCAAAAATAAGGGCCAATTCCTGGCTGTTATCTTGAAT

TCCTGATCTTTTTGACTTTCTGGGCCTCTAGGCACAGGAATTTTCCTCTTATCTTATCTA

GGAGTACAGTATAGAAAACAAGTTACCTACTCTGTCGCTTTCTTTCTGGACAAAGTCGCA

CTGCTACTGGTTCAGATCGGATCTCTACTGCTAGCTTAATCTGCAACCCAAGGGATGAAA

CTGAGAGCCAAATGCTGGAAACCAACCTTGGGTTGAGATGGATTGTAAAAGAGGAGAGTA

GGACGAGCAGGGGAAGCATTCCACTCTGCTACTAGCATACAGCAGGGCCATCTGCTTCTA

CTTGTGATGCAGCTACAGAATTCGGAAGGCAATTCTGGCAGCTCCGGCCAATCGCACGAA

GCAAGCAGAGAACGGAGCACGGTTGAGAAGACGCAGAGATCATCCCTTACCTCCCTCCGG

CTCCGGCCGACGGAAGCTGGGCGGAGCTTTTCTGGGTTTGAGGGCGCCTTCTTCCTTTTC

TCCTTCCCTCGAGCTCTCCTGCTCTGCTCAGCTGATCTCCTCCCTCTCCGCGTTGCCTCC

CTTTTTGTCGGCCCGAGCTGAGGGGCGGAGATGGAGACGCACGCACGCGCCACGCCGGAT

CTATCTCTACGTGTTGGTGCCGCGTGGGTTGAGCTGTGGAGGTCGCCCTGTTTTGACGTG

GCCGGCCCCACCCTGCCGGCGCAAGTTGGAACCCTTCGCCTCGCCGTTTTTGACCTTCCG

GCTCCGGAGGGGGCGCGAATCGACGTGGCGGGCTCAGGTCGGTTGGCCGCACCGCCGGTG

CACGCAGGCAAAGTCCAGCAGGGCTGCTGCTCCCGTTCTCAGATCAAGATCCAACGAAAA

CACGAGCGTCGAGCGGTGGTGTCGCCGCAGGGTGATCCGAGCTCGGGTTGTGTCGCCGCC

GCCGCGGCGGATCAGGCGATCTTTTTTTTTCCTATTCAAGTACGTAGTAGAAAAATGTGT

CGGCAGGCGTGTTAGCTGAATGAAATGCAACTGCGCAAGGAGGTGGTGCGGGCGTACGGC

ACGAACTAGAGAATCGGGATAACGTAGGGCCCAAATGTCACTCTCTCCTCGTGCCTTACC

TGAAAGCTCCAACCTCCGCCATCAAGAACGATG

***TaSpo11-1D* from Chinese Spring (TraesCS5D02G401100)**

>5D dna:chromosome chromosome:IWGSC:5D:466225339:466230635:1

CTGGTAGAGTTCCCGGTGGCGCAGCTCAATAGCAAGAGGTCCAGGCCTTCGACGAGCAAG

AGTAACTTCTGAATCCCGACAACGGTGTCCTCGAGATGTGAACCAGGGCCATTGATTGGG

CTGCGAAAAGAGCAAGCCCTTTGGGAGAGCTTAGCTGATGTATCATATGTGTAGCTGGGA

ACATGGGTTGTTCCCACCTTGATGGTTGAGGAGGAGCTTTTAGCTAGCTTAGCTGTTGTT

AGGTTTTTAGTGTAATTTTAACACGGCAACTCTACGAGTGAGCGATGAATAAAACTAAAA

ATATGTGCTTGTGTTTTTCGATCGATTCTCAGGTGGTTGAGAATTCAGACCTTCAGTCTG

ATTTGAGATTCTTGCTCTTCTGTTTTGAGGAAATTCTGTTTTTTCAGATATATATACTAC

ACTTTTTTTATTTTTTTTGTGAGGGGTTACTACACTTATATATGAAGAAAAGAAAATAAA

CCATGCGTTTTAAACAGGCCTTGTAGCAGAGGAGGCTTTCACAAAGCCCATTAGACACTA

GCCGTTACTGGACCAGTTGTGGCTTGTGACGGCGCTCTCCTGCTCTCCGAGCGACCGGTC

**ATGGCGGGGAGTGGGAAGCGGAGGCGCGCGACGGTGCTCGACGACGACGAGCGGCGGGGG**

**CGGCGGAGGCTGGAGGAGGCGGCCCTGCTCCTCCACAAGATCAAAG**GTGCGAATCCCCTT

CCCTCTCCTCGATCGACTGTAAACCCTCGTAATCCCCTTCGAATTGGGGTTGCAATCGGA

GTTAACGCGGTTTGCGCCGCCGCGCGCGTGGGCTGTCGGAGCAG**GGCTGGTGGGCTGGGT**

**GGTCGCGGAGATCAGCGCCGGCCGATCCCCTTCCCTGGCGCTCCACCGCTACCAGAACTA**

**CTGCGCCTCCGCCGACGCCGCCGCCGCCGCCGCGTCCCCGTCCACATG**GTTTGGCTCCCT

CCCTCCCTCTCTCGCCTTCAAATCTCTGCCGACCAAGCCTGTCAGATCCTCACCGGTGTG

TGCGCGTTCGCGGGGTGCGGGCGCGCGTGCAG**CGCCTGCAGCTACGACGCCCCCGTCGGC**

**ACGGACGTCCTCTCCCTCCTCCGCAAGGAATTCCACGCTTCCCGCCTCA**GTACGGATTCG

CGTACCCCAAACTGGCTCGATTCTCATCAATCTCTCGTTGTTTCTGATCATGCTAATCAG

TTCGAGATGCGTCGTTGATGTGCAG**ATGTGCTTCTCAGGGTCCTGCTCGTGGTGCAGCAA**

**CTCCTGCAGGAGAACAAGCACTGCTCCAAGAGGGACATCTATTACATGTACCCCTCCATG**

**TTCGTAG**GTCGGTGCTGGTCTGCGGTTGCCATGTCTGATCCATCTATGCGTAGTGTGAGT

TTCAGGTGTTCTGTTTCTTTGCTCTGTGTGTGACAATTACAAGTCTCACTGCAG**AACAAG**

**CGATTGTTGATCGTGCCATCAACGATATCTGCATACTCTTCAAGTGCAGCCGGCATAATC**

**TCAACGTG**GTATGTGCTACCGCTTGTCTGCCATCTTTGCAGAAGCTTTAGTTATGTGTTT

AGAAGTCATTTGTGCATTGGAGAATAAGCCTCTGGCTGTTATTTGATTATATGGTGTACA

ATGTGCCACACTGCGACTTGCTACTCCCTGGTTGTTCGTCCGTTATGGTGTACCACTGTC

CTATCTGATGTAGGATTATTTTCTTAATCTGTCTTTTGTGATGATTCCTTGAGGTCTTCA

TTTTTTCATGAATCGCTGGGAGCTGTTTCTGTTAGTTCTCTATAAGTGATTTCTACTGTA

TTTATTTCACCACCTCTTCACTTCTCTTTTAATAACCACCTACAATTTCTGCTCATTTGG

CTGTTTGCAG**GTTCCTGTGGCAAAAGG**GTATGGATTCTTATTGAGTTGTGAGTGTTTGAT

TTTGATAACATGTTTAATCCTTCCATCTTGTCCCATGCCGTTACTTAACTAATCACATTT

TTGTTCCTTCATCCTCCCTACCACTTGTCCTGCTTTACTTAAAACATGAGCTGGCTAAAC

AGTGGGATGCTGCACTTTTAGCAGACATTTAGAGCATCATAATCTCCTGTGTCCATACAA

TGGTCTTAGTTATTTCTGAACATTTTCCCGCTATGTACTCTTCAATGTCTTTATTGTTCC

AG**TTTGGTGATGGGCTGGATAAGATTTGTGGAGGGTGAAAAGAAAGTGTATTGTATAACA**

**AACGTCAATGCT**GTAAGCTTCTATGATTCTATCTATCTAATTACTCAATATGATCTGTTT

GTACTATGCATTAATTTGGCTAGCTTTTTTCCTTCTTCAAATGCAG**GCTTTCCCCATTCC**

**AGTTAGCATTGAGGCAATCAAAG**GTTGTTTAACAATTTGAAAACTTATAATAATCTTTGC

CATTTATAGATTTTTATTATTTCAAATCTGTTTCTTTAGGAAAATCAGTTTTAAAAATCG

TGATGTGCACAATTAGCTTATATCTCTTAGCAAGTCAATGTTCATTTTAAATTTTAATAG

ATAGTGATTGATGTATTAGGAAGTGGGATTTCGACAGACCACTAAATTTTGGACAGCATG

TGAAAGCGTAATGATAATTTATCTTTATTTTTTGTCTCAAACAAATATGGAGAGGAGTAT

CCTTGTTATTCCACAAACTATATGGCATAGGTTTGCTATCTTGTTATATGTACACTATAA

TTCTTTCATATAATATTCTCAATTGCTATGCAGTAAACTTAAGCTGTCTAAGTTTTTTCT

TGCAG**ATGTTGTTAGTGTTGCTCACTACATCCTTGTTGTTGAGAAGGAGGCAG**GTGTGAG

GATTAAAGGCCCCCTATCTGCATATGATTTTTCTATTCTTTGCAGTCTTCCTACAGTTTG

CTTCTGTATGCCATTTGCAG**TGTTCCAGCGTTTGGCCAATGACAAGTTCTGCGAAAAGAA**

**TCGCTGCATTGTTATTACA**GTAACTGCTAACGCTGCATCTAGTGTTATACTGCCATTATT

TTTCACATAGCATGTTTGATGGTTTTCTTCTGTTTACTCAG**GGAAGAGGCTACCCAGATA**

**TTCCAACAAGAAG**GCATGTGCTCTTTCGTATATTTGTTTCTCTTGTATATTAGAACTGAA

TTGCCATGTGAAACGTACATGCCTTGTAATGTTGCAG**ATTCTTGCGCTACCTTGTCGAAC**

**AGCTGCACTTGCCTGCTTATTGCTTAGTGGATTCAGATCCATATGGTTTTGATATTCTGG**

**CCACCTATAAATTTGGTTCCATG**GTAGGGCCAAAAATCTATCTAATATCTAATTATTTAT

TTGAAGTCCTAATAGCATCAAAGGCAGTGTAAATTTATCAGTGCAATTTCAATTTTTTTT

TGTTAAGCCAAGTGAAATGTGATTTTGTACATTTTATTCGAAATTTAGTCCCATTTTGTT

GGTATTTTTCTAGTGTTAACTGACCAACTCTTCCTGCTCCCTTGTCCAATTCTCATTCTA

TTTTAG**CAATTGGCATATGATGCAAATGTTCTGCGTGTGCCTGAGATACGATGGCTTGGG**

**GTCTTCACATCTGACTTTGAGGAGTATTGTCTTCCGGACTGCTGTCAATTACAATTGTCA**

**TCTGAAG**GTCACTAAATTGGAGGGATATAAATTTCATTTCTACTTCTTATCTCAGAAATT

GATTTGTTGTTAAATGTCAAATGCCATGTATCCTGCTTCAG**ATAGGAGGAAGCTTGAAGG**

**AATTCTCACCAGATGTTACTTGCACAAGGAAGCCCCAGAATGGAG**GTAAAGGAACAGGAA

GTTGTATTTCCTATTACGCGCATCCCAATAACTTTATGGCTATGATGTGGGTTGCAAGAA

TACTTCAAGTGCATCTAGAATTAAATAGAAGTTTTCACAATATCTTTCTTACAAAATGGC

AAATGCAACAGTTAAACTCAGTGATGTAAGTGAAATCTTCTTCCAAGCACAATGATCAAT

CCTATATTTAGGCCCTGTATTCCAAGCACTTCAGGGATATGATGTGGGTTGCAAATAATA

ATAGTTCGAGTCCAACTGCTAGAATTTGTTATTTGATTTGTTTGTTCTACTAGTAGTTAA

ATAGAGCTTTTCATATTTTGTTCTTACAAAATGTCAAATGCAACAACAAAACTCACTGAT

ATGAGTGAAATCTTCTTCCTGTACAATTATCAGTCTTATGATTTTATCCCGTGTAAGTAA

TCTGTAAGTGCTTTCAGCATCAATTCCTTAGTTTCGAAAACTCCATATCTGCGTCACCTA

GTCGACTCTTAACGGCAGTAGGTACTCCATTGTTCCAAAAGGTTTATGGCTGCTTTCACC

TGGTCAGTTCTAATGTCAACGGGTTTGTGTTTAAAATTAGAATCATTTGTGTAGTTAATA

GCCGGTTCTTTTTCCTGAAGATTTTTCTTCCTGAAATATTCATGACTATATAATCCTTGG

CAG**GTTGAAGTTGGAAGCCATGCTGGAAATGGGTGTCAAATTTGAGATTGAAGCATTGTC**

**AGCAAGTTCCATTTCGTTTCTGTCGCAAGAGTACATCCCCCAACAGATCAGACTAGGGAG**

**GTACATATAG**GATTGCATAGACCTGTATCTATGCAGAATTGTGCTAGGCTAAGAGTCTCC

TTTTGTTTTGGTTACACCACTTGTAGTTGCTTGTCTGATCAAACAATCACCTTGGTATTC

ACAACAAGAGTTTGACTGCAATTGGTGTATACCGTCAGTTACTGCATTGCTACTCATAAT

GCTTCTTGTTGTGCATAGATTAATTTGGGTTTATTATGTTAATGTGCAAAGGGCAACCTG

TTAGAAGTGAATGCCTGAATAGGGAACTCTGGATGTCCTCTTAGTTCTACCAGGTCACCA

TCTCATGGAAGCAAATTCCTATTTACCTGAGATCTCAGCCTCCACTTGCCGAGCATCATT

AGATATATTGTTGCTTGCCTTGCAATTCTGGAGCCAGAAGCACAAATCCAAGAAAAAAAT

ACATCTATACAGGCTACCATATTGGAAGCTGGAAATATTTCATGACTGGAAAATGGCTTA

ACAAACTTCATGTACACAGGCACATAAGCTTAGCGAAGCATCACCAAATACTTCTTTCTT

ACACGTGTCCAGGTGCATATGAGCAGATCCATTGTGTTAACTCAACAAGGTCGTATCTTT

TGACACTTTTACTAGATTACAGAATTAATTAACAGCATTAACTAACCATTCAACCATTTA

AGCTGTAACATTAAGTACAAGCCCATATGTTCGTCACTGACCGGATTGTGCCTGAGTAAG

GTCATATGTGGAGAGCG

**Figure S2.** Confirmation of Spo11-1 sgRNAs activity in wheat protoplasts. Wheat protoplasts were transformed with i) just the wheat optimised Cas9 construct (described by Zhang *et al*., 2019) or ii) with the Cas9 construct plus the SPO11-1 specific guide RNAs sg4 (5’GCACAGACCTACGAACATGG3’) and sg6 (5’GGAGAGGACGTCCGTGCCGA3’). **A.** High efficiency of wheat protoplast transformation as evidenced by a nuclear targeted, GFP reporter. **B.** Spo11-1 specific guide RNAs were designed to generate a deletion of ~200bp following amplification with primers designed to flank the region. Fragments were electrophoresed on a 2% agarose gel: (SS) New England Biolabs 1kb ladder; lane 1 is the negative control; lanes 2, 3 and 4 are from protoplasts transformed with both guide RNAs.  **C.** Aligned sequences of the larger (c. 500 bp) and smaller (c. 200 bp) bands after excision and sequencing; the sequences of the two guide RNAs are also shown.

A.


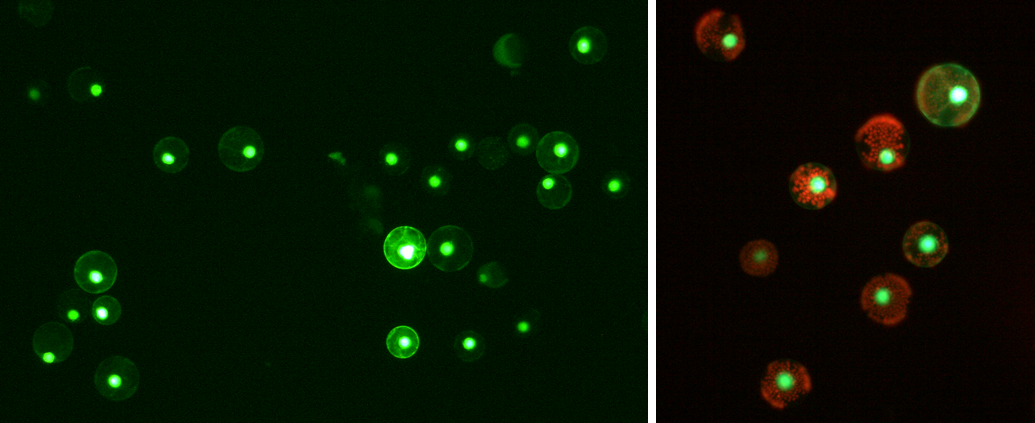


B.


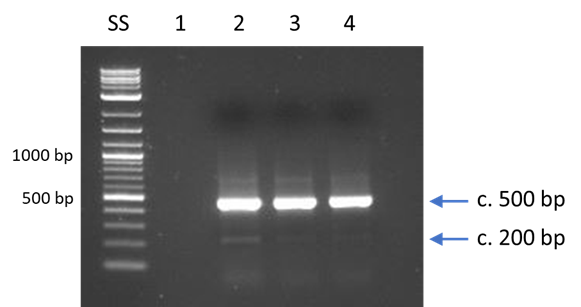


C.


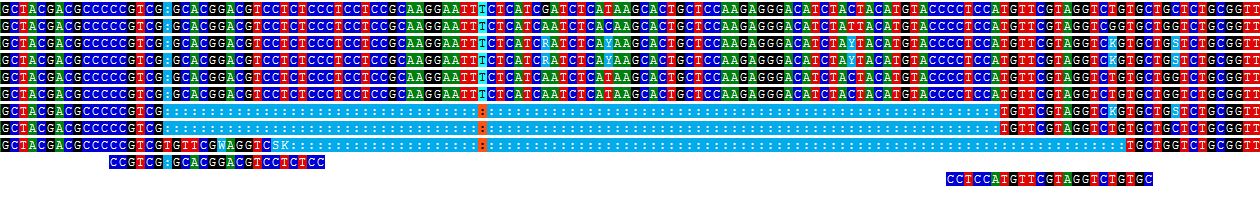


**Table S1**. Expected and observed genotypes of T1 progeny from plant 22

| **Genotype** | **Observed percentage (in 200)** | **Expected percentage (in 200)** |
| --- | --- | --- |
| *spo11-1a1* homozygote | 23 | 25 |
| *spo11-1a2* homozygote | 26 | 25 |
| *spo11-1b1* homozygote | 22 | 25 |
| *spo11-1b2* homozygote | 21 | 25 |
| *spo11-1d1* homozygote | 21 | 25 |
| Triple edited homozygote* | 20 | 25 |
